# Genome-wide association studies and genetic architecture of carcass traits in Angus beef cattle using imputed whole-genome sequences data

**DOI:** 10.1186/s12711-025-00970-6

**Published:** 2025-06-01

**Authors:** Hasan Baneh, Nikolay Elatkin, Laurent Gentzbittel

**Affiliations:** 1 Project Center for Agro Technologies, Skolkovo Institute of Science and Technology, Moscow, 121205 Russia; 2 LLC “Miratorg-Genetika”, Moscow, Russia

## Abstract

**Background:**

Carcass related traits are economically important traits for the beef industry, which affect quantity, quality and pricing of meat and farmers profitability. The current study was carried out to identify genomic regions associated with carcass traits including carcass weight (CW), marbling score (MS), rib-eye area (REA), and back fat thickness (BFT). Genome-wide association studies (GWAS) were performed using linear mixed models on 6,511,978 imputed whole genome sequence (WGS) variants in a population of 13,241 Angus beef cattle. The genetic architecture of the traits was evaluated based on the GWAS results.

**Results:**

With a threshold of p-value < 3.96 × 10^–7^, 842, 745, 340, and 101 SNPs located in 13 genomic regions were significantly associated with CW, MS, REA, and BFT, respectively. While the majority of the identified quantitative trait loci (QTL) were trait-specific, two QTLs with pleiotropic effect were identified, including a QTL on BTA7 (88.25–91.96 Mb) affecting CW, MS and REA, and a QTL on BTA20 (4.55–5.01 Mb) affecting CW and BFT. Several important genes are harbored by the detected QTLs, which can be considered potential candidate genes for carcass traits in Angus beef cattle. Our findings also showed that higher density panels are more powerful in GWAS, such that the signals on BTA6 affecting CW, and two signals on BTA17 and BTA18 affecting MS were not detectable using medium SNP array genotypes. The allele substitution effects and additive genetic variances of the imputed variants followed a bell-shaped and a scaled inverse chi-squared distribution, respectively. Among functional categories, missense variants had the highest allele substitution effects for CW, MS and BFT, while 3′ UTR variants had higher effects for REA, compared to other functional classes.

**Conclusions:**

Our findings highlight the power of using imputation to perform GWAS and provide some valuable information for a better understanding of the underlying genetic background and architecture of carcass traits in beef cattle.

**Supplementary Information:**

The online version contains supplementary material available at 10.1186/s12711-025-00970-6.

## Background

The main objective of the beef cattle production system is meat production, which is mainly determined by the carcass weight of slaughtered animals. In addition, meat quality traits are of economic importance, as they affect product pricing and consumer satisfaction [1]. This has motivated breeders to consider these traits in genetic improvement programs [2]. Since the phenotypic observations for these traits are typically recorded post-slaughter, genetic progress through pedigree-based evaluations (e.g., BLUP) relies heavily on the quality and depth of pedigree information, as well as the degree of relatedness between selection candidates and phenotyped animals. However, the moderate to high heritability and significant economic importance of these traits [3–6], combined with the use of indirect phenotyping methods such as ultrasound scanning, have enabled breeders to achieve genetic improvement through the application of selection indexes.

The advent of new genotyping technologies has provided an opportunity to identify the variants associated with these traits, which can be directly used in selection programs. Utilizing genome-wide SNP genotypes for selecting the best candidates, known as genomic selection, accelerates genetic improvement for these traits by increasing the accuracy of genetic evaluation and shortening the generation interval [5]. The accuracy of the prediction models used in genomic selection are influenced by the genetic architecture underlying the trait [7]. In this regard, Cole et al. [8] reported that there were considerable differences (6–8%) in prediction accuracy between models with different priors for some traits in dairy cattle. Therefore, knowledge on genetic architecture underlying the economically important traits will improve the genomic prediction efficiency in beef cattle breeding programs. Genome-wide association studies (GWAS) rely on linkage disequilibrium (LD) between the markers and causal variants. The higher-density SNP arrays are more powerful in scanning the genome [9, 10] and, therefore, the significant variants, QTLs and candidate genes are expected to be identified more accurately. Weng et al. [11] identified six novel QTL affecting growth and carcass traits using a high-density panel (770 K) compared to the lower density arrays (50 K) in Brangus beef cattle.

Whole-genome sequencing is an ideal approach for scanning the entire genome and identifying variants that may not be detected by SNP chip array-based GWAS [12, 13]. However, the sequencing costs have decreased dramatically in recent years, sequencing a large number of animals in commercial herds is still expensive [14]. Genotype imputation is the cost-effective and powerful method to extend the genotype information from a lower density array to the higher density panels and whole genome sequences. In this approach, a small number of animals are genotyped with a higher-density panel and used as the reference population to impute the SNP genotypes that are not directly genotyped by the lower density marker panel. This strategy has been applied to identify the variants affecting economically important traits in dairy and beef cattle, and in some cases, novel variants have been discovered [12, 13, 15]. Therefore, it is expected that applying higher-density genotypes (e.g., whole genome sequence data) result in unrevealing novel causal variants, candidate genes and genomic signals associated with quantitative traits. The main objective of this study was to identify novel genomic regions underlying carcass weight and meat quality traits in Angus beef cattle using genome-wide association studies based on imputed whole-genome sequence SNPs.

## Methods

### Population and phenotypes

The data used in this study were collected from a Black Angus beef cattle population belonging to Miratorg company. The animals were born in different farms that are geographically close and have very similar climate conditions. All the farms are genetically connected and operate under the same management system. Newborn calves remain with their mothers for 4–6 months, followed by pasture grazing until they reach 12–15 months of age and approximately 350 kg in weight. Subsequently, the calves will be kept in the feedlots for about 7 months.

In this study, the phenotypic and genotypic data from 13,241 Black Angus steers, born between 2017 and 2019 and reared in four different feedlots, were used. The animals were slaughtered at an average age of 700 days, between 2019 and 2021. After slaughtering, the studied traits were individually recorded by expert personnel using digital equipment. The traits considered for this study were carcass weight (CW, kg), rib-eye area (REA, in^2^), marbling score (MS, score) and back fat thickness (BFT, mm). CW was measured as hot carcass weight after slaughter. REA, MS and BFT were measured as the total area, marbling grade and the thickness of external fat, respectively, of the ribeye muscle (*longissimus dorsi*) between the 12th and 13th ribs. All traits were recorded for all individuals. The descriptive statistics of the studied traits are presented in Table 1.

**Table 1:** Descriptive statistics of the traits

Traits	No. records	Min	Max	Mean	SD	CV
CW	13,241	288.8	721.1	404.06	37.76	9.35
REA	13,241	6.91	16.65	11.87	1.33	11.20
MS	13,241	2.68	10.240	7.26	1.35	18.60
BFT	13,241	0.064	1.976	0.731	0.26	35.57

*CW* carcass weight, *REA* rib-eye area, *MS* marbling score, *BFT* Backfat thickness

### Genotypes and imputation

The genotyped animals were from 50 farms, with an average of 264 samples per farm (ranging from 57 to 1706). Genotyping was conducted using the Illumina Bovine 50 K SNP chip array (Illumina, San Diego, CA). All samples had a call rate higher than 0.90 and were therefore retained in the dataset. Duplicate markers, multiallelic sites, unmapped SNPs, and those located on mitochondrial or sex chromosomes were removed. In addition, SNPs with a minor allele frequency (MAF) < 0.05 or a SNP call rate < 0.95 were excluded. In total, 39,580 SNPs located on autosomal chromosomes for 13,241 samples were used for downstream analysis. Quality control was carried out using PLINK v1.07 [16]. The SNP chip genotypes were imputed to Whole Genome Sequence (WGS) level using 128 Angus samples of the “1000 Bull Genomes Project” sequence data [9] as the reference population, which is public freely accessible (project ID: PRJEB42783; Accession: ERP001736). Only bi-allelic variants (n = 13,123,690 SNPs) were retained for further steps. In order to impute the genotypes with high accuracy, a pilot study was conducted to determine optimum filtration thresholds of sequences in reference population and to calibrate the input parameters of the program. The imputation accuracy for each scenario was calculated based on concordance occurrence rate, which was measured as the percentage of correctly imputed genotypes out of 5000 randomly selected masked SNPs. We found that imputation accuracy was strongly affected by the sequence quality and the allele frequency of variants in the reference population. Therefore, the reference dataset was filtered based on MAF > 0.02, sequence depth > 3, sequence quality > 30 and missing rate < 20%. After filtering, a total of 9,268,297 autosomes SNPs remained and were used as the reference panel. Genotype imputation was performed using Beagle v4.1 [17]. The input parameters of the program were calibrated, and those with the highest accuracy were chosen. In addition, the pedigree-based genotype imputation approach did not improve imputation accuracy, most likely due to incompleteness and shallowness of the pedigree. Therefore, the population-based genotype imputation algorithm implemented in Beagle was applied. The imputed genotypes were filtered based on Dosage R-Squared (DR^2^ ≥ 0.8), resulting in 6,544,904 SNPs with an average DR^2^ of 0.946 and a range of 0.81 to 1. In addition, the imputed SNPs with a MAF < 0.01 were excluded and, consequently, 6,511,978 SNPs were retained for GWAS analyses. Descriptive statistics of marker interval of the imputed WGS panel over the *Bos taurus* autosomes (BTA) are provided in Additional file 1: Table S1.

### Genome-wide association studies (GWAS)

Population stratification was assessed using principal component analysis (PCA) performed by GCTA v1.93.3 program [18] applying on 50 K SNPs chip array genotypes. Genome-wide association studies (GWAS) were performed using a single-SNP mixed linear model implemented in GCTA v1.93.3 program [18]. The statistical model for estimating the SNP effects was as follows:

$${\mathbf{y}} = {\mathbf{Xb}} + {\mathbf{s}}{\text{s}} + {\mathbf{Zu}} + {\mathbf{e}}$$

where **y** is the vector of observation for the studied traits including CW, REA, MS and BFT; **b** is the vector of fixed effects; s is the SNP effect, **s** is a vector that contains the genotypes where the homozygote of the first allele (SS), the heterozygote (Ss), and the homozygote of the second allele (ss) was coded as 0, 1, 2, respectively; **u** is the vector of polygenic effects; and **e** is the vector of random residuals. **X**, **S** and **Z** are incidence matrices relating **b** and **u**, respectively. Fixed effects included birth year, birth month, recording year, recording month, birth farm, feedlots and age at recording (as covariate). It is assumed that **u** is normally distributed as **u** ∼ *N* (**0**, **G** ${\upsigma }_{\text{a}}^{2}$). **G** is the additive genomic relationship matrix constructed using 50 K SNP chip genotypes, following the method proposed by Yang et al. [18], where ${\upsigma }_{\text{a}}^{2}$ is the genetic variance explained by genome-wide SNPs. In addition, it was assumed that **e** is normally distributed as **e** ∼ *N* (**0**, $\mathbf{I}{\upsigma }_{\text{e}}^{2}$), where **I** is the identity matrix and ${\upsigma }_{\text{e}}^{2}$ is the residual variance. The explained genetic variance (EGV) by each SNP was calculated as:

$$EGV_{i,j} \left( \% \right) = \frac{{2p_{i} q_{i} \beta_{i,j}^{2} }}{{{\upsigma }_{{a_{j} }}^{2} }}{*}100,$$

where p_i_, q_i_ are the allele frequencies of SNP i, β_i,j_ is the effect of marker i on trait j, and ${\upsigma }_{{a}_{j}}^{2}$ is the additive genetic variance of trait j. Genetic architecture of the studied traits was investigated by plotting allele substation effects (β) and additive genetic variance ($2{p}_{i}{q}_{i}{\beta }_{i}^{2}$) for all the 6.5 M variants, as well as the distribution of β for large-effect SNPs. SNPs with large effects were defined as those beyond the extremes of the distribution of β, represented as $\overline{\beta } \pm \gamma *{\sigma }_{\beta }$ where, $\overline{\beta }$ and ${\sigma }_{\beta }$ are the mean and standard deviation of SNP effects for the trait, respectively, and $\gamma$ ranges from 1 to 5.

### Significant threshold

To avoid type-I error and consequently false-positive results, the p-values were adjusted according to the Bonferroni correction for multiple testing procedure. The common procedure for medium-density chip arrays $\left( {\frac{{\text{p - value threshold}}}{{{\text{No}}.{\text{ SNPs}}}}} \right)$ is very conservative and may result in false-negative associations [19]. Therefore, the genome wide significant threshold was defined as ${\text{p - value}}_{{{\text{adj}}}} = \frac{0.05}{{{\text{N}}_{{{\text{eff}}}} }},$ where ${\text{N}}_{\text{eff}}$ is the effective number of SNPs (${\text{N}}_{\text{eff}}$ = 126,415). The effective number of SNPs was calculated as the number of independent SNPs (not in LD with ${r}^{2}>0.5$), in a 5000 bp window of the genome and sliding 500 SNPs, using PLINK v1.07 [16].

### Variant annotation

The functional annotation of the imputed sequence variants was performed using the Variant Effect Predictor (VEP) online web interface [20], by mapping on Ensembl (release 106) annotation of the *Bos taurus* bovine genome assembly ARS-UCD1.2. Since the functional annotations described in Ensembl are classified into many and usually overlapping classes, we categorized the functions into nine groups: (1) Intergenic region variants, (2) Downstream gene variants, (3) Upstream gene variants, (4) Synonymous variants, (5) Intron variants, (6) Missense variants, (7) 3′ UTR variants, (8) 5’ UTR variants, and (9) Other regulatory region variants. The last group included variants of splice regions in introns and disruptive inframe deletion. The candidate genes harboring the significant SNPs, were identified by intersecting SNP positions (± 100 Kb) with the *Bos taurus* genome assembly (ARS-UCD1.2) using BEDTools v. 2.30.0 [21]. In addition, the significant SNPs were compared to the cattle QTLs in the Animal QTLdb database [22]. GWAS results of each trait were visualized by plotting (− log10) p-values against SNP positions in Manhattan plots. Quantile–quantile (Q–Q) plots were also generated to assess the distribution of observed p-values relative to the expected distribution under the null hypothesis, providing insight into the goodness of fit and potential inflation or deflation of test statistics. The visualizations were performed using the R packages qqman [23] and ggmanh [24].

## Results

**Fig. 1:**
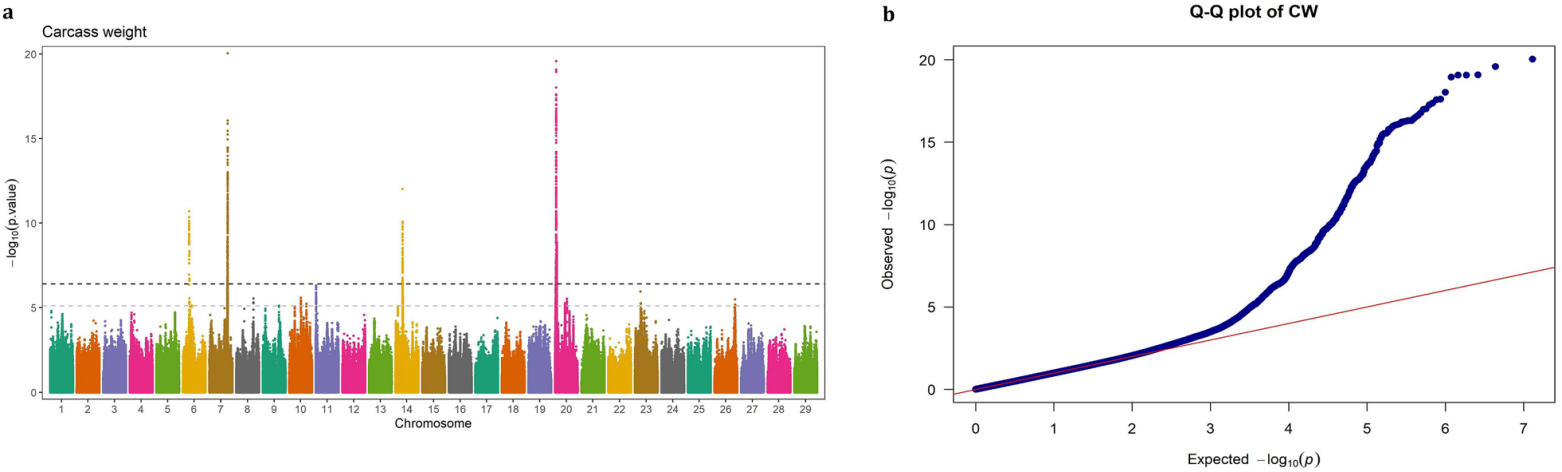
GWAS for carcass weight using imputed WGS variants. **a** Manhattan plot, **b** QQ plot

**Fig. 2:**
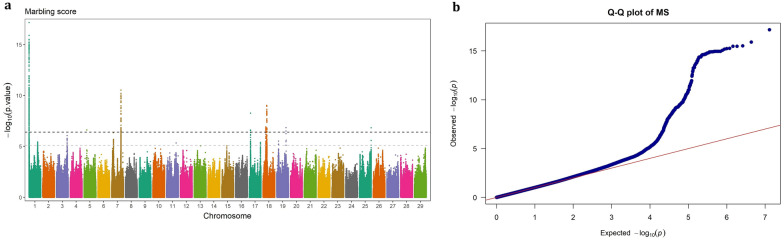
GWAS for marbling score using imputed WGS variants. **a** Manhattan plot, **b** QQ plot

**Fig. 3:**
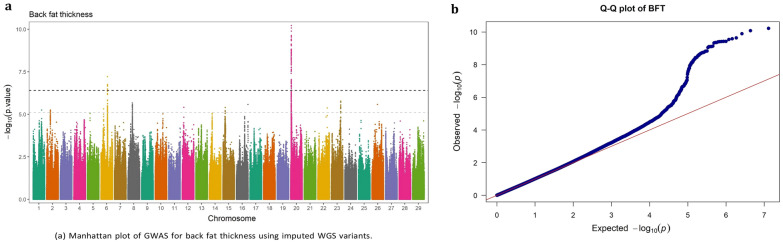
GWAS for back fat thickness using imputed WGS variants. **a** Manhattan plot, **b** QQ plot

**Fig. 4:**
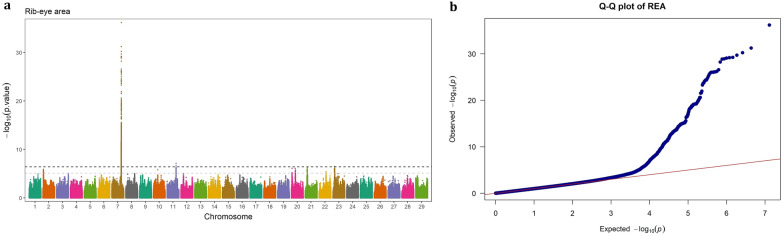
GWAS for rib-eye area using imputed WGS variants. **a** Manhattan plot, **b** QQ plot

**Fig. 5:**
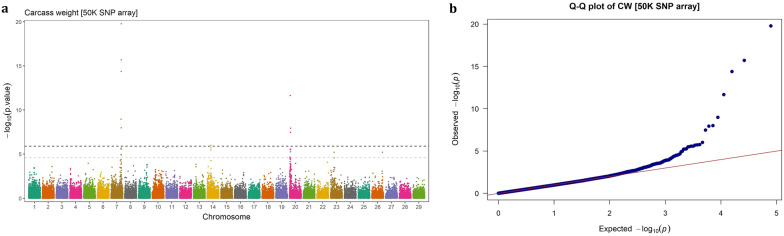
GWAS for carcass weight using 50 k SNP chip array genotypes. **a** Manhattan plot, **b** QQ plot

**Fig. 6:**
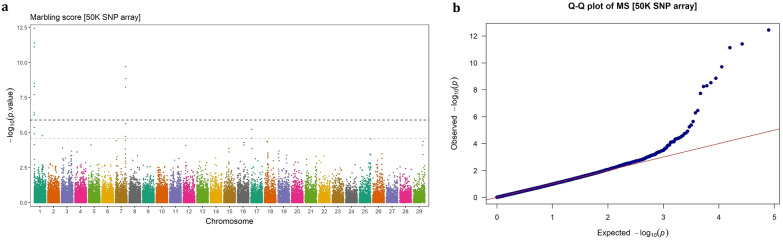
GWAS for marbling score using 50 k SNP chip array genotypes. **a** Manhattan plot, **b** QQ plot

**Fig. 7:**
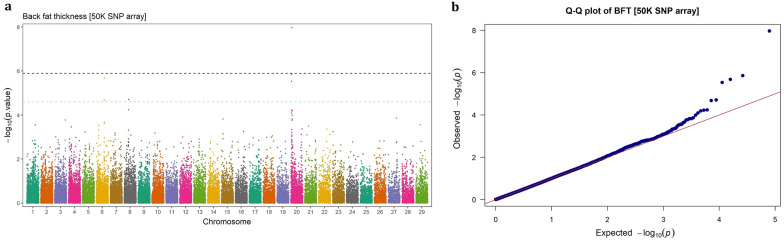
GWAS for back fat thickness using 50 k SNP chip array genotypes. **a** Manhattan plot, **b** QQ plot

**Fig. 8:**
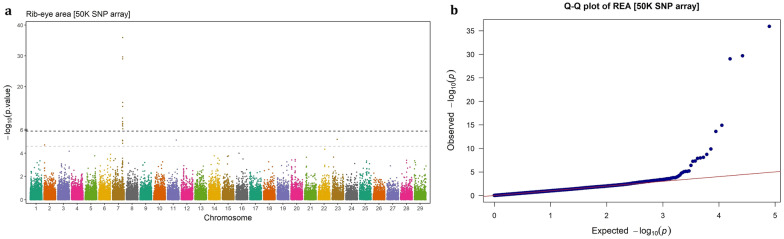
GWAS for rib-eye area using 50 k SNP chip array genotypes. **a** Manhattan plot, **b** QQ plot

The Manhattan and QQ plots of GWAS results based on imputed WGS variants and 50 K SNP array genotypes for CW, REA, MS and BFT are presented in Figs. 1, 2, 3, 4, 5, 6, 7, 8, respectively. The QQ-plots did not show any potential inflation or deflation in the results of genome-wide association studies when using both medium-density SNP chip arrays and imputed WGS data. Principal component analysis revealed no evidence of structure stratifications in the studied population (data not shown).

### Functional annotation variant effects

The distribution of the SNPs over the nine functional annotations classes showed that > 90% of the SNPs were located in intergenic and intron regions (see Additional file 2: Table S2). The effects of SNPs of each functional class on the traits were evaluated by the average of squared allele substitution effect of the variants belonging to that class. The missense variant category exhibited the highest average and variance of the SNP effects, and explained additive genetic variances for CW and MS. In contrast, for BFT and REA, variants in 5ʹ UTR region showed the highest contribution to genetic variance. Nonetheless, the missense and 3′ UTR variants had relatively high effects on BFT and REA, respectively (see Additional file 3: Table S3).

### Genetic architecture of the traits

The allele substitution effects of imputed WGS variants were normally distributed (bell-shaped) with means around zero across all studied traits, in which the majority of SNPs had small effects, while only a small proportion of SNPs had large effects. For all traits, the plots of additive genetic variance of the variants followed a scaled inverse chi-square distribution (see Additional file 4: Figure S1 to Additional file 7: Figure S4). Notably, the distribution of estimated SNP effects was consistent across the traits, such that > 73% of the variants falling within one standard deviation (SD) around of the mean allele substitution effect (see Additional file 8: Figure S5). Furthermore, the SNPs with extreme effects (mean ± 3 × SD) represented about 1% of the total variants.

### Imputed WGS vs. SNP chip array

The results showed that higher resolution of the genotypes leads to detection of novel genomic regions, which are not detectable using a lower dense array. In this study, several signals were identified using imputed WGS, which were not observed when using 50 K SNP chip genotypes. A novel region on BTA6 (at 38.09–37.05 Mb) containing 48 significant SNPs associated with CW (Fig. 1) and two regions located on BTA17 (5.397–5.398 Mb) and BTA18 (15.33–19.41 Mb), harboring 3 and 41 significant SNPs, respectively, associated with MS were only evident with the imputed WGS data (Fig. 3).

### Genome-wide association results

In this study, 1,697 imputed SNPs distributed across 12 autosomal chromosomes reached the significant threshold (p-value < 3.96 × 10^–7^) (see Additional file 9: Figure S6). The distribution of the significant genomic regions over the chromosomes differ among the traits. For instance, significant SNPs associated with BFT were found exclusively on two chromosomes, while the SNPs associated with MS were spread over seven different autosomes. Additionally, the number of significant SNPs ranged from 842 SNPs for CW to 101 SNPs for BFT. We also identified 745 and 340 significant SNPs significantly associated with REA and MS, respectively. About 98% (n = 1,675) of significant SNPs were not included in the Bovine 50 K SNP chip array (see Additional file 10: Table S4). Moreover, 17% of the significant SNPs (n = 289) were found to be associated with at least two traits, suggesting the presence of genes with pleiotropic effects in these regions. Of those, 42 SNPs located on BTA7 were associated with CW, MS and REA. Additional common significant variants among the traits included 156 SNPs on BTA7 (CW and REA), 2 SNPs on BTA7 (MS and REA), and 89 SNPs on BTA20 (CW and BFT) (see Additional file 11: Figure S7).

### SNP effects and mapped genes

The SNP positions (± 100 Kb) were mapped on the *Bos taurus* genome assembly to explore potential candidate genes. Among the SNPs, 483 SNPs did not intersect with any annotated genes, which were the intergenic variants located more than 100 Kb away from the annotated genes. The comparison of unmapped SNPs with those located within (or flanking) the genes revealed that unmapped SNPs, on average, had lower effects, genetic variance, and explained genetic variance of CW, MS, and REA. In addition, those associated with CW and MS had a little higher minor allele frequency (see Additional file 3: Table S3). There was only one significant unmapped SNP for BFT (Table 2).

The chromosomal position of the significant genomic regions, along with the MAF range, p-value and EVG of the SNPs within the region, and the top significant SNP (referred to as the “lead SNP”) in each region, are summarized in Table 2. A large proportion of the significant SNPs (~ 79.26%) were located in non-genic regions including intergenic regions (n = 1184), upstream (n = 38) and downstream (n = 123) of annotated genes. The potential candidate genes associated with CW, MS, BFT and REA are given in Tables 3, 4, 5, 6, respectively. In addition, for each lead SNP, the position, MAF, p-value, and explained genetic variance by the SNP is provided.

**Table 2:** Genome regions harboring the significant SNPs associated with the traits, along with top significant SNP in each region

Trait	BTA^1^	No. SNP	Region (Kb)	Length (Kb)	MAF	p-value	EVG^2^ (%)	Top significant SNP				
								Position	Allele	MAF	p-value	EVG (%)
CW	6	20	37,048.59–38,094.56	1045.97	0.01–0.02	4.45 × 10^–10^–2.47 × 10^–07^	0.89–1.27	37,573,615	A/T	0.01	4.45 × 10^–10^	1.27
CW	7	201	89,109.92–91,966.66	2856.74	0.17–0.49	9.11 × 10^–21^–3.82 × 10^–07^	1.15–4.55	90,819,463	T/A	0.40	9.11 × 10^–21^	4.55
CW	14	108	22,899.09–23,800.49	901.41	0.01–0.04	9.38 × 10^–13^–3.93 × 10^–07^	0.88–1.66	22,988,388	C/T	0.02	9.38 × 10^–13^	1.66
CW	20	513	3068.86–7077.16	4008.3	0.20–0.50	2.58 × 10^–20^–3.92 × 10^–07^	1.14–4.95	4,967,158	A/G	0.38	2.58 × 10^–20^	4.95
MS	1	247	526.97–2108.12	1581.15	0.11–0.43	6.95 × 10^–18^–2.98 × 10^–07^	0.61–2.38	1,083,284	A/G	0.27	6.95 × 10^–18^	2.38
MS	5	1	26,334.86–26,334.86	–	0.03–0.03	2.35 × 10^–07^–2.35 × 10^–07^	0.58–0.58	26,334,859	G/A	0.03	2.35 × 10^–07^	0.58
MS	7	45	90,524.96–91,636.03	1111.07	0.24–0.43	2.91 × 10^–11^–3.94 × 10^–07^	0.83–1.66	90,689,453	A/G	0.38	2.91 × 10^–11^	1.66
MS	17	3	5397.62–5398.34	0.72	0.43–0.49	5.38 × 10^–09^–2.82 × 10^–07^	0.94–1.22	5,398,343	T/C	0.43	5.38 × 10^–09^	1.22
MS	18	41	15,326.95–19,412.92	4085.98	0.21–0.34	9.44 × 10^–10^–3.71 × 10^–07^	0.84–1.37	18,147,371	G/A	0.23	9.44 × 10^–10^	1.37
MS	19	2	50,534.79–50,536.06	1.27	0.27–0.27	1.40 × 10^–07^–3.10 × 10^–07^	0.95–1.04	50,534,794	T/A	0.27	1.40 × 10^–07^	1.04
MS	25	1	41,405.81–41,405.81	–	0.34–0.34	1.46 × 10^–07^–1.46 × 10^–07^	0.81–0.81	41,405,807	A/G	0.34	1.46 × 10^–07^	0.81
FAT	6	9	69,122.35–69,128.33	5.98	0.30–0.31	6.16 × 10^–08^–3.79 × 10^–07^	1.86–2.10	69,122,406	A/G	0.31	6.16 × 10^–08^	2.10
FAT	20	92	4546.40–5203.39	656.99	0.30–0.46	6.03 × 10^–11^–3.17 × 10^–07^	1.92–3.25	4,978,475	T/C	0.38	6.03 × 10^–11^	3.25
REA	7	741	88,254.56–92,008.12	3753.56	0.03–0.50	6.43 × 10^–37^–3.93 × 10^–07^	0.78–7.99	90,819,463	T/A	0.40	6.43 × 10^–37^	7.99
REA	11	2	80,216.88–80,286.58	69.71	0.46–0.49	6.92 × 10^–08^–2.29 × 10^–07^	1.11–1.19	80,286,584	C/T	0.46	6.92 × 10^–08^	1.19
REA	23	2	7830.73–7831.94	1.21	0.05–0.05	3.10 × 10^–07^–3.32 × 10^–07^	1.14–1.15	7,830,730	A/G	0.05	3.10 × 10^–07^	1.15

^1^*BTA* Bos taurus autosomes, ^2^*EGV* explained genetic variance

**Table 3:** Potential candidate gene intersected by the significant SNPs associated with carcass weight, along with the lead SNP

Ensembl Gene ID	Gene symbol	Biotype^1^	BTA	No. SNPs	Variants^2^	Lead SNP							
						Position	Allele	Variant^2^	Distance (bp)	MAF	p-value	│β│ ± SE	EVG (%)
*ENSBTAG00000005989*	*LAP3*	PCG	6	1	Intg	37,048,588	A/G	Intg	92,164	0.01	1.77 × 10^–09^	11.80 ± 1.96	1.18
*ENSBTAG00000005932*	*FAM184B*	PCG	6	6	Intron	37,188,623	A/G	Intron	Within	0.01	9.14 × 10^–10^	12.33 ± 2.01	1.23
*ENSBTAG00000046561*	*LCORL*	PCG	6	9	Intr, Intg	37,573,615	A/T	Intg	16,509	0.01	4.45 × 10^–10^	12.51 ± 2.01	1.27
*ENSBTAG00000006031*	*ADGRV1*	PCG	7	97	Intr, Down, Intg	90,672,235	A/G	Down	4426	0.40	8.51 × 10^–17^	4.29 ± 0.52	3.59
*ENSBTAG00000007116*	*ARRDC3*	PCG	7	10	Intr, Down, Intg	90,819,463	T/A	Intg	20,102	0.40	9.11 × 10^–21^	4.83 ± 0.52	4.55
*ENSBTAG00000044050*	*XKR4*	PCG	14	21	Intr, Intg	22,988,388	C/T	Intg	34,617	0.02	9.38 × 10^–13^	11.76 ± 1.65	1.66
*ENSBTAG00000005893*	*TMEM68*	PCG	14	14	Intg	22,995,750	A/T	Intg	38,530	0.03	1.39 × 10^–08^	6.72 ± 1.18	1.19
*ENSBTAG00000020034*	*LYN*	PCG	14	22	3ʹUTR,Down,Intg	23,247,588	G/A	Down	2836	0.03	2.88 × 10^–07^	6.15 ± 1.20	0.95
*ENSBTAG00000019147*	*RPS20*	PCG	14	7	Intg	23,268,723	A/C	Intg	9593	0.03	3.66 × 10^–07^	6.08 ± 1.20	0.94
*ENSBTAG00000028889*	*U1*	snRNA	14	1	Up	23,289,158	C/T	Up	4609	0.03	2.53 × 10^–07^	6.05 ± 1.17	0.96
*ENSBTAG00000019145*	*MOS*	PCG	14	8	Down, Intg	23,312,227	C/T	Intg	12,028	0.02	2.40 × 10^–10^	10.21 ± 1.61	1.29
*ENSBTAG00000004022*	*PLAG1*	PCG	14	9	Intr, Down	23,329,375	C/T	Down	1166	0.02	1.56 × 10^–10^	10.30 ± 1.61	1.32
*ENSBTAG00000049910*	*CHCHD7*	PCG	14	3	3ʹUTR,Down,Intg	23,384,445	C/T	Down	1312	0.02	4.51 × 10^–10^	9.93 ± 1.59	1.25
*ENSBTAG00000054153*		PCG	14	1	Down	23,392,938	G/T	Down	2885	0.02	6.53 × 10^–10^	9.80 ± 1.59	1.23
*ENSBTAG00000018570*	*SDR16C5*	PCG	14	3	Down, Intg	23,421,387	A/G	Intg	7017	0.02	8.23 × 10^–11^	9.73 ± 1.50	1.37
*ENSBTAG00000040321*	*SDR16C6*	PCG	14	1	Intg	23,467,842	G/C	Intg	8100	0.02	1.75 × 10^–10^	10.62 ± 1.66	1.32
*ENSBTAG00000043923*	*U6*	snRNA	14	15	Intg	23,778,696	A/C	Intg	36,809	0.02	1.11 × 10^–09^	9.40 ± 1.54	1.17
*ENSBTAG00000024801*	*RANBP17*	PCG	20	1	Intr	3,068,857	G/T	Intr	Within	0.22	1.74 × 10^–07^	2.88 ± 0.55	1.14
*ENSBTAG00000015316*	*NPM1*	PCG	20	1	Up	3,204,915	G/C	Up	678	0.26	1.58 × 10^–07^	2.73 ± 0.52	1.18
*ENSBTAG00000009019*	*SH3PXD2B*	PCG	20	38	Intg	4,298,909	C/T	Intg	75,518	0.47	1.41 × 10^–09^	2.97 ± 0.49	1.79
*ENSBTAG00000001429*	*NEURL1B*	PCG	20	2	Intr, Intg	4,470,843	T/G	Intg	22,372	0.32	2.96 × 10^–11^	3.67 ± 0.55	2.38
*ENSBTAG00000013863*	*DUSP1*	PCG	20	1	Up	4,546,402	G/A	Up	2791	0.30	1.69 × 10^–13^	4.18 ± 0.57	3.01
*ENSBTAG00000015955*	*ERGIC1*	PCG	20	34	Intr,3ʹUTR,Down, Intg	4,654,475	T/C	Intr	Within	0.37	1.68 × 10^–17^	4.77 ± 0.56	4.29
*ENSBTAG00000015099*	*RPL26L1*	PCG	20	4	Up	4,716,043	A/G	Up	942	0.42	1.31 × 10^–14^	4.15 ± 0.54	3.41
*ENSBTAG00000015100*	*ATP6V0E1*	PCG	20	15	Up, Intr, Down, Intg	4,728,635	T/C	Down	3889	0.42	7.95 × 10^–15^	4.09 ± 0.53	3.32
*ENSBTAG00000002020*	*CREBRF*	PCG	20	4	Intg, Up, Intr	4,771,537	C/G	Up	4976	0.50	1.95 × 10^–12^	3.58 ± 0.51	2.61
*ENSBTAG00000002021*	*BNIP1*	PCG	20	8	Intr,3ʹUTR,Intg	4,847,839	G/C	Intr	Within	0.44	1.34 × 10^–10^	3.39 ± 0.53	2.31
*ENSBTAG00000050983*	*5S_rRNA*	rRNA	20	6	Down, Intg	4,901,496	A/G	Down	3291	0.45	1.62 × 10^–10^	3.37 ± 0.53	2.29
*ENSBTAG00000020568*	*NKX2-5*	PCG	20	2	Down, Intg	4,907,590	G/T	Down	2576	0.45	1.43 × 10^–10^	3.38 ± 0.53	2.3
*ENSBTAG00000048827*		lncRNA	20	26	Up, Down, Intg	4,928,741	T/C	Down	4292	0.41	3.22 × 10^–14^	4.13 ± 0.54	3.37
*ENSBTAG00000001823*	*STC2*	PCG	20	52	Intr, Miss, Down, Intg	4,967,158	A/G	Intg	32,567	0.38	2.58 × 10^–20^	5.10 ± 0.55	4.95
*ENSBTAG00000034598*	*BOD1*	PCG	20	16	Intr, Down, Intg	5,312,377	C/A	Intg	7141	0.45	2.95 × 10^–12^	3.43 ± 0.49	2.37
*ENSBTAG00000049048*		lncRNA	20	1	Up	5,333,774	C/T	Intr	Within	0.40	1.71 × 10^–07^	2.50 ± 0.48	1.22
*ENSBTAG00000009995*	*CPEB4*	PCG	20	1	Up	5,603,903	T/C	Up	2162	0.20	3.52 × 10^–07^	3.03 ± 0.60	1.18
*ENSBTAG00000016002*	*FAM169A*	PCG	20	2	Intg	6,653,722	A/G	Intg	23,075	0.32	4.59 × 10^–08^	3.27 ± 0.60	1.87
*ENSBTAG00000015519*	*GFM2*	PCG	20	1	Intr	6,762,158	T/C	Intr	Within	0.31	1.45 × 10^–08^	3.41 ± 0.60	2.03
*ENSBTAG00000034138*		PCG	20	1	Intr	6,841,524	G/C	Intr	Within	0.31	2.07 × 10^–09^	3.62 ± 0.60	2.29
*ENSBTAG00000026369*	*ENC1*	PCG	20	211	Intg, Intr, Syn	6,971,591	C/T	Intg	40,803	0.33	1.10 × 10^–10^	3.78 ± 0.59	2.55

^1^*PCG* Protein-coding gene, ^2^*Intg* Intergenic, *Intr* Intron, *Down* Downstream, *Up* Upstream, *Miss* Missense, *Syn* Synonymous

**Table 4:** Potential candidate gene intersected by the significant SNPs associated with marbling score, along with the lead SNP

Ensembl Gene ID	Symbol	Biotype^1^	BTA	No. SNPs	Variants^2^	Lead SNP							
						Position	Allele	Variant^2^	Distance (bp)	MAF	p-value	│β│ ± SE	EVG (%)
*ENSBTAG00000053686*	*5S_rRNA*	rRNA	1	2	Intg	529,512	A/C	Intg	49,977	0.14	8.58 × 10^–11^	0.165 ± 0.025	1.19
*ENSBTAG00000001753*	*–*	PCG	1	12	Intr, Down, Intg	809,296	G/A	Intr	Within	0.14	1.13 × 10^–12^	0.178 ± 0.025	1.41
*ENSBTAG00000001150*	*KCNE1*	PCG	1	1	Up	1,039,894	T/G	Up	629	0.14	5.61 × 10^–10^	0.160 ± 0.026	1.09
*ENSBTAG00000026259*	*–*	PCG	1	6	Intg, Up, Down	1,063,932	C/T	Down	2335	0.15	1.98 × 10^–10^	0.157 ± 0.025	1.14
*ENSBTAG00000051226*	*–*	PCG	1	11	Intg,Up,5′ UTR, Down	1,083,284	A/G	Intg	6040	0.27	6.95 × 10^–18^	0.181 ± 0.021	2.38
*ENSBTAG00000013841*	*C21orf140*	PCG	1	64	Intg, Down	1,091,439	C/A	Intg	7688	0.27	1.72 × 10^–14^	0.161 ± 0.021	1.89
*ENSBTAG00000011528*	*SMIM11A*	PCG	1	17	Down, Intr	1,109,692	T/C	Intr	Within	0.26	9.09 × 10^–16^	0.171 ± 0.021	2.07
*ENSBTAG00000026260*	*KCNE2*	PCG	1	82	Intg, Up, Intr, Miss, Down	1,146,986	T/C	Intg	15,238	0.35	3.07 × 10^–16^	0.165 ± 0.020	2.25
*ENSBTAG00000054497*	*–*	lncRNA	1	1	Intg	1,279,506	G/C	Intg	96,075	0.13	2.94 × 10^–07^	0.120 ± 0.023	0.61
*ENSBTAG00000012594*	*MRPS6*	PCG	1	3	Intr	1,395,323	T/C	Intr	Within	0.28	1.79 × 10^–15^	0.152 ± 0.019	1.71
*ENSBTAG00000018278*	*ATP5PO*	PCG	1	1	Intg	1,587,635	C/T	Intg	55,993	0.21	5.27 × 10^–08^	0.114 ± 0.021	0.79
*ENSBTAG00000008490*	*CRYZL1*	PCG	1	4	Intr	1,929,664	G/C	Intr	Within	0.22	3.47 × 10^–12^	0.162 ± 0.023	1.61
*ENSBTAG00000009187*	*DNAJC28*	PCG	1	1	Down	2,030,767	G/C	Down	725	0.22	2.09 × 10^–12^	0.162 ± 0.023	1.65
*ENSBTAG00000043399*	*–*	snoRNA	1	7	Up	2,093,032	C/T	Up	3224	0.42	1.72 × 10^–10^	0.127 ± 0.020	1.44
*ENSBTAG00000012899*	*IFNGR2*	PCG	1	24	Intr, Syn, Down	2,096,344	T/C	Down	2624	0.40	3.37 × 10^–11^	0.131 ± 0.020	1.5
*ENSBTAG00000015016*	*CALCOCO1*	PCG	5	1	Intr	26,334,859	G/A	Intr	Within	0.03	2.35 × 10^–07^	0.218 ± 0.042	0.58
*ENSBTAG00000006031*	*ADGRV1*	PCG	7	37	Intr, Down, Intg	90,689,453	A/G	Intg	21,644	0.38	2.91 × 10^–11^	0.139 ± 0.021	1.66
*ENSBTAG00000007116*	*ARRDC3*	PCG	7	1	Intg	90,819,463	T/A	Intg	20,102	0.40	1.81 × 10^–10^	0.134 ± 0.021	1.59
*ENSBTAG00000007953*	*FBXW7*	PCG	17	3	Intg	5,398,343	T/C	Intg	46,909	0.43	5.38 × 10^–09^	0.117 ± 0.020	1.22
*ENSBTAG00000002980*	*GPT2*	PCG	18	6	Intr, Intg	15,361,890	C/A	Intg	12,697	0.25	1.19 × 10^–07^	0.114 ± 0.022	0.9
*ENSBTAG00000003757*	*DNAJA2*	PCG	18	9	Intg	15,363,043	C/G	Intg	12,123	0.26	1.46 × 10^–07^	0.113 ± 0.022	0.89
*ENSBTAG00000017397*	*ZNF423*	PCG	18	10	Intr	18,147,371	G/A	Intr	Within	0.23	9.44 × 10^–10^	0.146 ± 0.024	1.37
*ENSBTAG00000007942*	*TENT4B*	PCG	18	1	Down	18,650,478	A/G	Down	4713	0.32	3.71 × 10^–07^	0.104 ± 0.021	0.87
*ENSBTAG00000006208*	*ADCY7*	PCG	18	7	Intr, Up,	18,652,008	C/T	Up	3899	0.29	6.21 × 10^–09^	0.123 ± 0.021	1.14
*ENSBTAG00000021575*	*BRD7*	PCG	18	7	Intg, Intr	18,773,905	A/G	Intg	6912	0.32	2.23 × 10^–09^	0.126 ± 0.021	1.26
*ENSBTAG00000028470*	*5S_rRNA*	rRNA	18	1	Intg	19,412,923	G/T	Intg	90,313	0.21	2.07 × 10^–07^	0.126 ± 0.024	0.96
*ENSBTAG00000050786*	*–*	PCG	19	2	Up	50,534,794	T/A	Up	Within	0.27	1.40 × 10^–07^	0.119 ± 0.023	1.04
*ENSBTAG00000019303*	*MAFK*	PCG	25	1	Down	41,405,807	A/G	Down	300	0.34	1.46 × 10^–07^	0.099 ± 0.019	0.81

^1^*PCG* Protein-coding gene, ^2^*Intg* Intergenic, *Intr* Intron, *Down* Downstream, *Up* Upstream, *Miss* Missense, *Syn* Synonymous

**Table 5:** Potential candidate gene intersected by the significant SNPs associated with back fat thickness trait, along with the lead SNP

Ensembl Gene ID	Symbol	Biotype^1^	BTA	No. SNPs	Variants^2^	Lead SNP							
						Position	Allele	Variant^2^	Distance (bp)	MAF	p-value	│β│ ± SE	EVG (%)
*ENSBTAG00000020658*	*LNX1*	PCG	6	9	Intr	69,122,406	A/G	Intr	Within	0.31	6.16 × 10^–08^	0.022 ± 0.004	2.1
*ENSBTAG00000013863*	*DUSP1*	PCG	20	1	Up	4,546,402	G/A	Up	2791	0.30	1.55 × 10^–07^	0.021 ± 0.004	2.02
*ENSBTAG00000015955*	*ERGIC1*	PCG	20	16	Intr	4,665,088	A/G	Intr	Within	0.36	7.91 × 10^–10^	0.025 ± 0.004	2.95
*ENSBTAG00000015099*	*RPL26L1*	PCG	20	3	Up	4,713,237	T/G	Up	3748	0.42	2.85 × 10^–09^	0.023 ± 0.004	2.67
*ENSBTAG00000015100*	*ATP6V0E1*	PCG	20	4	Intr, Down, Intg	4,753,160	A/G	Intr	Within	0.45	1.43 × 10^–07^	0.020 ± 0.004	2.05
*ENSBTAG00000002020*	*CREBRF*	PCG	20	2	Intg, Intr	4,812,157	C/T	Intr	Within	0.45	2.39 × 10^–07^	0.020 ± 0.004	1.98
*ENSBTAG00000002021*	*BNIP1*	PCG	20	7	Intr,3ʹUTR,Intg	4,854,589	T/C	3_prime_UTR	Within	0.45	8.90 × 10^–08^	0.020 ± 0.004	2.12
*ENSBTAG00000050983*	*5S_rRNA*	rRNA	20	4	Down, Intg	4,883,602	C/A	Intg	14,485	0.45	1.02 × 10^–07^	0.020 ± 0.004	2.1
*ENSBTAG00000020568*	*NKX2-5*	PCG	20	2	Down, Intg	4,904,957	A/C	Intg	5209	0.45	1.94 × 10^–07^	0.020 ± 0.004	2.01
*ENSBTAG00000048827*	*–*	lncRNA	20	11	Up, Down, Intg	4,927,232	G/A	Down	2783	0.41	8.45 × 10^–11^	0.025 ± 0.004	3.22
*ENSBTAG00000001823*	*STC2*	PCG	20	41	Intr, Miss, Down, Intg	4,978,475	T/C	Intg	21,250	0.38	6.03 × 10^–11^	0.026 ± 0.004	3.25

^1^*PCG* Protein-coding gene, ^2^*Intg* Intergenic, *Intr* Intron, *Down* Downstream, *Up* Upstream, *Miss* Missense, *Syn* Synonymous

**Table 6:** Potential candidate gene intersected by the significant SNPs associated with rib-eye area trait, along with the lead SNP

Ensembl Gene ID	Symbol	Biotype^1^	BTA	No.SNPs	Variants^2^	Lead SNP							
						Position	Allele	Variant^2^	Distance (bp)	MAF	p-value	│β│ ± SE	EVG (%)
*ENSBTAG00000020701*	*MEF2C*	PCG	7	84	Intg, Up, Intr	88,400,182	C/T	Intr	Within	0.30	1.81 × 10^–11^	0.141 ± 0.021	1.94
*ENSBTAG00000003258*	*–*	PCG	7	2	Intg	88,572,721	G/A	Intg	30,366	0.21	7.14 × 10^–09^	0.150 ± 0.026	1.70
*ENSBTAG00000048981*	*–*	lncRNA	7	74	Intg	88,904,278	G/A	Intg	55,931	0.32	1.85 × 10^–10^	0.132 ± 0.021	1.75
*ENSBTAG00000046692*	*bta-mir-3660*	miRNA	7	4	Intg	89,493,888	G/A	Intg	64,215	0.24	2.89 × 10^–10^	0.156 ± 0.025	2.04
*ENSBTAG00000050847*	*–*	PCG	7	1	Intg	89,871,499	T/C	Intg	58,679	0.24	2.06 × 10^–10^	0.158 ± 0.025	2.08
*ENSBTAG00000014373*	*CETN3*	PCG	7	1	Intr	89,953,020	C/A	Intr	Within	0.23	7.57 × 10^–12^	0.176 ± 0.026	2.52
*ENSBTAG00000013612*	*MBLAC2*	PCG	7	1	Intr	90,026,124	C/A	Intr	Within	0.23	1.84 × 10^–12^	0.181 ± 0.026	2.66
*ENSBTAG00000044077*	*POLR3G*	PCG	7	1	Intr	90,074,590	G/C	Intr	Within	0.23	2.50 × 10^–12^	0.178 ± 0.025	2.61
*ENSBTAG00000006031*	*ADGRV1*	PCG	7	152	Intg, Up, Intr, splices, Miss, Syn, Down	90,672,235	A/G	Down	4426	0.40	5.76 × 10^–32^	0.247 ± 0.021	6.82
*ENSBTAG00000007116*	*ARRDC3*	PCG	7	35	Intr, Down, Intg	90,819,463	T/A	Intg	20,102	0.40	6.43 × 10^–37^	0.268 ± 0.021	7.99
*ENSBTAG00000042596*	*7SK*	misc_RNA	7	13	Intg, Up	91,814,590	C/T	Intg	14,455	0.34	4.90 × 10^–09^	0.119 ± 0.020	1.47
*ENSBTAG00000023928*	*RDH14*	PCG	11	1	Intg	80,216,879	C/T	Intg	51,362	0.49	2.29 × 10^–07^	0.098 ± 0.019	1.11
*ENSBTAG00000054726*	*UQCC2*	PCG	23	2	Intr	7,830,730	A/G	Intr	Within	0.05	3.10 × 10^–07^	0.233 ± 0.046	1.15

^1^*PCG* Protein-coding gene, ^2^*Intg* Intergenic, *Intr* Intron, *Down* Downstream, *Up* Upstream, *Miss* Missense, *Syn* Synonymous

### Carcass weight

Four genomic regions located on chromosomes 6, 7, 14, and 20, harboring 20, 201, 108, and 513 SNPs, respectively, were found to be significantly associated with CW. The SNPs located on BTA6 (37.05–38.09 Mb) were novel variants, as they were not detected in the GWAS based on 50 K SNP chip array genotypes. Although these SNPs were highly significant (p-value < 2.47 × 10^–7^) with strong effects on CW, due to their low minor allele frequency (0.01 ≤ MAF < 0.023) their contribution to the genetic variance of the trait was relatively small (0.09–1.28%). The top SNP in this region (BTA6: 37,573,615), explaining ~ 1.28% of the total genetic variance, was located at 16.51 Kb away from the *LCORL* gene. This region also overlapped with two other genes, *LAP3* and *FAM184B*. In this region, two SNPs (BTA6: 37,048,588 located 92.16 Kb away from *LAP3* and BTA6: 37,188,623 located within *FAM184B*), were identified, which explained 1.18% and 1.23% of the genetic variance of CW, respectively.

The significant SNPs located at ~ 89.11 to 91.97 Mb on BTA7 were found to be highly important for CW, as they explained a relatively large proportion (ranging from 1.15% to 4.55%) of the genetic variance of the trait, and exhibited high MAF (0.169 ≤ MAF ≤ 0.487). This genomic region was mapped to the *ADGRV1* and *ARRDC3* genes.

The significant SNPs located on BTA14 (n = 108) fall into a 90.41 Kb region (22.90–23.80 Mb) and were mapped on the 10 protein-coding genes, including *XKR4*, *TMEM68*, *LYN*, *RPS20*, *MOS*, *PLAG1*, *CHCHD7*, *SDR16C5*, *SDR16C6*, and two snRNA genes (*ENSBTAG00000028889* and *ENSBTAG00000043923*). However, the genes are well-known for their involvement in growth in cattle, they explained less than 1.8% of genetic variance of CW in this population, most likely due to low MAF (< 0.04).

We also identified 513 significant SNPs on BTA20 distributed over a ~ 4 Mb genomic region (~ 3.07–7.08 Mb). These SNPs mapped to both within (intron, exon; missense and synonymous) or flanking regions (intergenic, upstream, downstream and 3ʹ UTR) of the 18 annotated protein-coding genes (*RANBP17*, *NPM1*, *SH3PXD2B*, *NEURL1B*, *DUSP1*, *ERGIC1*, *RPL26L1*, *ATP6V0E1*, *CREBRF*, *BNIP1*, *NKX2-5*, *STC2*, *BOD1*, *CPEB4*, *FAM169A*, *GFM2*, *ENSBTAG00000034138*, *ENC1*) and 3 non-coding genes (*5S_rRNA*, *ENSBTAG00000048827*, *ENSBTAG00000049048*). These SNPs were relatively highly polymorphic in the studied population (MAF = 0.2–0.5) and the genetic variance they explained for CW were in a range of 1.14 to 4.95%, highlighting the importance of this QTL in contributing to the genetic variation of the trait.

### Marbling score

The significant SNPs associated with marbling score, were distributed over several autosomes, including BTA1 (n = 247), BTA5 (n = 1), BTA7 (n = 45), BTA17 (n = 3), BTA18 (n = 41), BTA19 (n = 2), and BTA25 (n = 1). In general, the significant SNPs located on BTA1 had a greater effect on MS compared to those on the other chromosomes (EGV ranging from 0.61 to 2.38% vs. 0.58 to 1.66%). The three most significant SNPs were BTA1: 1,083,284 (6.04 Kb away from *ENSBTAG00000051226*), BTA1: 1,146,986 (15.24 Kb away from *KCNE2*), and BTA1: 1,109,692 (an intron variant of *SMIM11A*), which explained 2.38, 2.25 and 2.07% of the genetic variance in MS, respectively.

The identified QTL on BTA18, spanning ~ 15.33 to 19.41 Mb, was a novel genomic region associated with MS, such that was not detectable using the Bovine 50 K SNP chip array. It was the longest genome region associated with MS in this study, encompassing 41 relatively high polymorphic variants (MAF > 0.21) with relatively large effects on the trait (EVG: 0.84%–1.37%). The most significant SNP within this region (BTA18: 18,147,371, EVG = 1.37%) is an intron variant of the *ZNF423* gene, which plays an important role in adipogenesis in cattle. Also, *KCNE2*, another biologically relevant gene involved in lipid metabolism, intersected with 82 significant SNPs, among which a missense variant (BTA1: 1,124,522) with allele substitution effect 0.159 ± 0.03 score.

In total, we identified 24 potential candidate protein-coding genes associated with MS in Angus beef cattle, as listed in Table 4. Among these, *ADGRV1* and *ARRDC3* genes seem to have pleiotropic effects, as SNPs located within or near these genes were significantly associated with CW and REA as well. In addition, we identified four non-coding RNA genes, including two ribosomal RNA genes, one long non-coding RNA (lncRNA) and one small nucleus RNA (snoRNA) gene. Seven significant SNPs were mapped on a snoRNA gene (*ENSBTAG00000043399*), from which the lead SNP located 3.22 Kb away from the gene, explained 1.44% of the genetic variance for MS. Notably, this QTL was not detected using 50 K SNP array genotypes, encompasses several important candidate genes involved in lipid metabolism, such as *DNAJA2* and *ZNF423* genes.

### Back fat thickness

The significant SNPs associated with BFT were located on BTA6 (n = 9) and BTA20 (n = 92). All the identified SNPs had high MAF (> 0.30) with relatively large effects, each explaining ≥ 1.86% of the genetic variance for BFT in this population. All the nine significant SNPs on BTA6 were intron variants within the protein-coding *LNX1* gene. The significant SNPs on BTA20 fall into a 656.99 Kb genomic region (4.55–5.20 Mb), intersecting with *DUSP1*, *ERGIC1*, *RPL26L1*, *ATP6V0E1*, *CREBRF*, *BNIP1*, *ENSBTAG00000050983*, *NKX2*-5, *ENSBTAG00000048827*, and *STC2* genes. The top significant SNP in this region, BTA20: 4,978,475, is an intergenic variant, located 21,250 bp away from *STC2* gene, which accounted for 3.25% of the genetic variance. Interestingly, the second most significant SNP, BTA20:4,927,232 (EVG = 3.22%), was located 2.78 Kb downstream of the long non-coding RNA *ENSBTAG00000048827* gene.

### Rib-eye area

Three genome regions, located on BTA7 (88.25 to 92.01 Mb), BTA11 (80.22 to 80.29 Mb), and BTA23 (7830.73 to 7831.94 Kb), were identified to be associated with REA in Angus beef cattle. The regions on BTA11 and BTA23 exhibited similar effects on the trait, (EGV ≈ 1.1%), though they had considerably different MAFs (0.4 vs. 0.05, respectively). The region on BTA7 contained 741 SNPs with a wide range in both MAF (0.025–0.498) and EGV (0.78%–7.99%). Among these, two SNPs (BTA7:90,819,463 and BTA7:90,672,235) were extremely significant (p-value < 6.43 × 10^–37^ and p-value < 5.76 × 10^–32^, respectively) and explained a large proportion of the genetic variance (7.97% and 6.82%, respectively). The first SNP is an intergenic variant, located 20.1 Kb away from *ARRDC3* gene, while the second is located in the downstream region of *ADGRV1* gene (4.43 Kb away). These two genes were significant for the CW and MS traits as well. The *ADGRV1* gene was surrounded by 152 significant intergenic, upstream, intron, splice, exon and downstream variants. Apart from these two genes, the SNPs significant for REA were mapped to 8 other protein-coding genes, a long non-coding RNA gene, a micro-RNA (*bta-mir-3660*) gene and a misc-RNA gene.

## Discussion

Carcass related traits are economically important traits in beef cattle production system, which have attracted much attention in breeding programs. In this study, carcass weight, marbling score, rib-eye area and back fat thickness traits were investigated for GWAS and genetic architecture in Angus beef cattle. It has been reported that sample size is the main determinant factor in GWAS power [25], particularly for rare variants [26]. In addition, population stratification can lead to spurious associations GWAS [27]. By accounting for polygenic effects in the model, the absence of stratification effects in the studied population and relatively large sample size (> 13,000), the applied single-marker mixed linear model fitted the data properly (QQ-plots).

The results indicated that the denser genotype panels enhance not only the resolution of detected signals but also the power to identify novel genomic regions in association studies. In this study, the imputed WGS led to detection of three novel genome regions that were not identified using 50 K SNP array genotypes, including a region associated with CW on BTA6 (37.05–38.09 Mb) and two regions associated with MS on BTA17 (at 5.39 Mb) and BTA18 (15.33–19.41 Mb). Similarly, Weng et al. [11] identified six novel QTLs using a high-density SNP chip (770 K) compared to a lower density panel (50 K) in Brangus beef cattle. In another study, Wang et al. [12] showed that imputed WGS-based GWAS identified additional significant loci for carcass merit traits in multiple Canadian beef cattle populations, compared to the 50 K SNPs array genotypes. Since GWAS rely on linkage disequilibrium (LD) between markers and causal variants, higher-density marker genotypes such as imputed WGS, will scan the genome more deeply and increase the likelihood of capturing variants that are not in LD with markers on low-density arrays. Therefore, denser panels are expected to be more powerful in detecting the signals and, consequently, offering deeper insights into the genetic architecture of complex traits [9].

In this study, a total of 1697 SNPs distributed over 12 autosomes reached the significance threshold. The majority of these significant variants were located in intergenic regions (69.77%), while only 0.53% were missense or synonymous variants. Although the intergenic regions were not previously well known and were considered “junk DNA”, but they are now recognized for their potential roles in transcription regulation, particularly through epigenetic mechanisms such as CpG islands (de)methylation [28]. Intersecting the significant SNPs with the *Bos taurus* genome assembly indicated that, 483 intergenic variants are located more than 100 Kb away from any annotated genes in the cattle genome. These SNPs had lower average substitution effects on the traits, but slightly higher MAF, compared to the other SNPs.

A total of 13 QTLs were identified for carcass weight and meat quality traits in Angus beef cattle. Of those, 11 QTLs (comprising 1,408 SNPs; 83%) were trait-specific, while two QTLs showed potential pleiotropic effects. The latter QTLs were located on BTA7 (65 genic and 135 intergenic SNPs) affecting CW, MS and REA and on BTA20 (28 genic and 61 intergenic SNPs) affecting CW and BFT. The remaining SNPs (n = 1214) were mapped within or in proximity (< 100 Kb) on 64 protein-coding and 12 non-coding RNA genes (e.g., lncRNA, rRNA, snRNA, miRNA, snoRNA and miscRNAs) based on the current *Bos taurus* genome assembly. A genomic region on BTA1 (spanning 526.97–2108.12 Kb and containing 247 SNPs) associated with MS intersected with 15 potential candidate genes. This region has previously been reported to be associated with MS, weaning weight [29] and residual feed intake [30]. This QTL is considered an important region for MS because of its enrichment of several genes involved in lipid metabolism pathways. The lead SNP in this region, an intergenic variant located only 6 kb away from the *ENSBTAG00000051226* gene, explained 2.25% of genetic variance. This gene, along with *KCNE1*, *SMIM11A*, *KCNE2,* and *ENSBTAG00000026259*, has been previously reported to be associated with milk fat percentage [14]. In addition, Hou et al. [31] reported that the *KCNE2* gene is differentially expressed during lactation in Holstein dairy cattle, probably contributing to milk fat percentage changes during the lactation. Among the other candidate genes of this region, *KCNE1*, *KCNE2*, *SMIM11A*, and *MRPS6* have been reported to be linked to body weight in Blanco Orejinegro beef cattle [32].

*MRPS6* is a key potential candidate gene for MS, harboring an intron variant (at 1,395,323 bp), which explained 1.71% of the genetic variance. This gene has been reported to be differentially expressed between groups with high and low MS [33]. There is no report on association of *CRYZL1* with MS. However, this gene has been reported to be linked with polledness [34] and sperm mortality [35]. Another gene in this region is *DNAJC28*, a member of the *DNAJ* gene family, which plays an anti-apoptotic role, an important function for meat tenderness [36]. In addition, the association of *DNAJC28* gene with feed efficiency in Angus cattle [37] and adaptability [38–40] has been reported.

The interferon gamma receptor 2 gene (*IFNGR2*) encodes the non-ligand- binding beta chain of the gamma interferon receptor, which is involved in immunological pathways. While some studies have suggested that mutations in *IFNGR2* may influence polledness status, Stafuzza et al. [41] stated that this gene had no known function related to horn growth. However, its potential role in intramuscular fat metabolism remains unclear. In our study, we identified 12 significant SNPs intersecting with the *chloride intracellular channel 6* (*CLIC6*) gene, including an intron variant that explained 1.41% of the genetic variance for MS. Despite this significant effect, no previous studies have reported the role of *CLIC6* in MS, and no overlapping QTLs were found in Animal QTL Database [22]. To the best our knowledge, this is the first report indicating an association of CLIC6 with an economic trait in beef cattle.

A significant SNP was identified on BTA5 at ~ 26 Mb for MS, an intron variant of the *Calcium Binding and Coiled-Coil Domain 1* (*CALCOCO1*) gene, which explained a relatively small proportion of the genetic variance of the trait (~ 0.58%), most likely due to its low MAF (0.034). Schrooten et al. [42] previously reported a QTL on BTA5 (at 35 cM) affecting milk fat yield in Holstein dairy cattle. Moreover, this region (BTA5:26 Mb) has been reported to be associated with MS [29] and trans-12-C18:1 fatty acid content in Angus beef cattle [43]. The *CALCOCO1* gene provides a link between phosphate and glucose metabolism, protein synthesis and degradation, calcium signalling, and cell growth [44]. It is also involved in a gene network that may influence meat quality traits in cattle [45].

The genomic region on BTA6 at 37–38 Mb, associated with CW, mapped on three key growth-related genes (*LAP3*, *FAM184B* and *LCORL*). This region has been previously reported to influence several traits, including body weight and post weaning daily gain [15, 46, 47], dry matter intake and metabolic body weight [15], carcass weight [1, 12, 48, 49], and bone weight [50]. In addition, Weng et al. [11] identified a QTL (at 38 Mb), which accounted for 6.95% of the genetic variance of birth weight in Brangus beef cattle.

*LAP3*, as a member of *LAPs* family, is involved in the control of oxytocin hydrolysis [1] and plays important role in maturation, inactivation, and degradation of proteins [51], as well as cell maintenance and growth development [13, 52]. The *LCORL* gene encodes a transcription factor that is believed to regulate the gene(s) involved in growth and or appetite [53]. Previous studies have highlighted the critical role of these genes in various traits, for example, growth and development in cattle [54], and sheep [55], carcass traits [10, 53, 56], and calving ease [57]. Therefore, *LAP3* and *LCORL* may be considered positional candidate genes for growth and carcass weight in beef cattle.

Another genomic region on BTA6 (at 69.12 Mb) associated with BFT was identified. This region has been previously reported to affect the internal fat weight and percentage [58], milk fat yield and percentage [59], as well as subcutaneous fat thickness [60]. The significant SNPs (n = 9) identified within region are intron variants of *LNX1* gene, which is involved in the ubiquitination pathway. This pathway is known to influence post-mortem meat quality [61] and tenderness [62].

A QTL on BTA7 (88.25–92.01 Mb) was detected using both the 50 K SNP chip genotypes and imputed WGS variants; however, the signal of the later was considerably sharper. To confirm that this peak reflects real associations rather than being driven by a large LD block in this population, we conducted additional association analyses. To do, the leading SNP (BTA7:90,819,463), located in the coding region of *ARRDC3* gene, was included as an additional fixed effect in the model. As a result, the significance level (p-value) of the associations with CW and MS drastically decreased to below the threshold, while some SNPs associated with REA remained significant (see Additional file 12: Figure S8). Furthermore, including the first 3 PCs as covariates in the model did not alter the significance level of the SNPs (see Additional file 13: Figure S9), suggesting that the observed signal is unlikely to be due to any stratified structure in this population.

The results revealed that this genomic region has pleiotropic effects on CW, MS and REA. Interestingly, the effects of the variants on CW were in the opposite direction of their effect on REA. This region has previously been reported to affect body weight at various ages, CW, BFT, REA, and MS in Angus cattle [29, 63, 64], Brangus cattle [11], and multi-breed and crossbred populations [65]. In addition, grilled beef flavour intensity has been reported to be influenced by this QTL [66]. This genomic region is of particular interest of beef cattle breeders, as it affects traits that are highly relevant to profitability. In the present study, this QTL harbored 745 significant variants, which were mapped on several potential candidate genes. These genes are involved in various mechanisms and aspects of growth and metabolism, which partially explain the pleiotropic nature of this QTL.

*MEF2C* is a member of the *MEF2* family, which plays a pivotal role in the morphogenesis and myogenesis of skeletal, cardiac, and smooth muscle cells [67]. The *MEF2C* gene is selectively expressed in differentiated myocytes and activates the transcription of nearly all skeletal and cardiac muscle genes [68]. In a study, Hu et al. [69] demonstrated that the *bta-miR-23a* can promote myogenic differentiation of fetal bovine skeletal muscle-derived progenitor cells by post-transcriptional downregulation of *MDFIC* gene. Moreover, *MDFIC* can regulate *MEF2C* transcriptional activity by regulating *MyoG*. The *bta-mir-3660*, has been suggested to play an important role in the growth by interacting with a QTL affecting calf size [70]. The *CETN3* and *ADGRV1* genes have been reported to contribute to rear teat placement [71] and coat color [72] in cattle. Another potential candidate gene is the *ARRDC3*, a member of the arrestin superfamily, which is probably involved in obesity [73, 74], growth [15], and adipose tissue development [64]. On BTA11, a single significant variant (at 80.22 Mb) associated with REA reached the significance threshold, which is 51.36 Kb away from the *RDH14* gene. McClure et al. [29] reported this region corresponds to the longissimus muscle area, body weight and MS. Another trait-specific QTL on BTA14, spanning 22.89–23.80 Mb, was identified, containing 108 significant SNPs associated with CW. Previous studies have reported that this QTL affects several economically important traits in cattle, including average daily gain [75], yearling weight [76], metabolic body weight [15], and CW [12, 64].

Our findings confirm the previously published reports indicating that this genomic region harbors several well-known genes which might be considered as potential candidate genes for carcass traits in Angus cattle. For instance, *PLAG1*, as a member of the *PLAG* gene family, is recognized as a key regulator of mammalian growth [77]. The activation of its product, which is a zinc finger transcription factor, results in up-regulation of genes such as *IGF-II*, thereby promoting cell proliferation and overall growth [37]. The *LYN* gene, as another example, encodes a tyrosine protein kinase that regulates cell proliferation, differentiation, apoptosis, migration and metabolism [78]. The other genes within this region, notably *XKR4*, *TMEM68*, *RPS20* and *CHCHD7* have been described to be implicated in various traits in cattle, such as growth [49, 64], age at puberty [79], body weight and growth [2, 80], intramuscular fat [81], CW [49], meat tenderness [82] and REA [77].

The significant SNPs (n = 3) on BTA17, located at a relatively short region (5397.62–5398.34 Kb), were found to be related to MS. The region has been previously reported to be associated with body weight and growth traits [29] and as a part of a larger QTL for MS [83]. The *FBXW7* gene, harboring these significant SNPs, has been recently reported to be associated with birth weight [6]. We also identified a 4 Mb region on BTA18 (15.33–19.41 Mb) harboring 41 variants affecting MS. Morris et al. [84] reported this region affects palmitic acid content of the *longissimus dorsi* muscle, while Spurlock et al. [85] reported its association with feed intake in dairy cattle. The *ZNF423* gene, has been associated with carcass merit and meat quality traits [52]. *ZNF423* influences adipocytes differentiation by regulating *PPARγ* expression [86]. Furthermore, *ZNF423* is targeted by *BTA-miR23a*, a miRNA that negatively regulates the adipocyte differentiation and lipid accumulation by targeting ZNF423 [87], which could potentially impact intramuscular fat. Another gene in this region, *DNAJA2*, a member of the *DNAJ* gene family, plays a crucial role in meat tenderness due to its anti-apoptotic properties [36].

In this study, one SNP on BTA19 (at 50 Mb) reached the threshold, which is located in a wider region affecting MS [29]. It has been reported that this region influences fatty acid composition in subcutaneous adipose fat, milk fat [88], and milk fat yield [60].

A QTL on BTA20 (at 3–7 Mb) with pleiotropic effects on CW and BFT was identified. Similarly, Morris et al. [58] reported that this region affects internal fat weight and CW. In addition, previous studies have suggested that this region underlie body weight and daily gain [63, 65, 76], dry matter intake, metabolic body weight [15], and CW [12]. This QTL harbors several potential candidate genes, notably *RANBP17*, *SH3PXD2B*, *NEURL1B*, *DUSP1*, *CREBRF*, *STC2,* and *CPEB4*, which ate involved in a wide range of biological functions.

The *RANBP17* gene is involved in diverse biological processes, including cell growth, differentiation, and development [89, 90]. The *SH3 and PX domain 2B* (*SH3PXD2B*) protein is essential for normal postnatal development [91]. Li et al. [92] reported that the *NEURL1B* gene affects *longissimus dorsi* muscle area in Hanwoo cattle. *DUSP1*, also known as *MKP-1*, contributes to the *mitogen-activated protein kinase* (*MAPK*) cascade, which is involved in various cellular functions such as cell proliferation, differentiation, and migration [64]. Minster et al. [93] identified a mutation in *CREBRF* that increases fat storage in adipocyte cells and strongly influences body mass index in Samoans. The *STC2* gene has been implicated in cell proliferation in various cancers [94], postnatal growth [95], bone development and skeletal muscle growth in mice [96], as well as adiposity and obesity in nondiabetic humans [97]. *CPEB4* is related to the biological process related to growth such as proliferation of muscle and bones as well as adipogenesis [98]. The genes located in this genomic region play functional roles in pathways related to both growth and adiposity, supporting the pleiotropic behavior of this QTL.

The allele substitution effects of the significant SNPs identified within this QTL were not in the same direction for CW and BFT. However, this inverse relationship aligns with the breeding objective of increasing CW and reducing BFT. In this study, two SNPs on BTA23, located at 7,830,730 and 7,831,938 bp, were found to be significantly associated with REA. McClure et al. [29] reported that a QTL on BTA23 (at 7 Mb) is associated with mature and carcass weight in cattle. However, there is no report on relationship of this region with REA. On BTA25, a SNP (BTA25: 41,405,807) affecting MS reached the significant threshold. To our knowledge, this is the first report associating this region with MS in beef cattle. Previous studies have reported a QTL on BTA25 at ∼ 40 Mb, to be associated with residual feed intake [30], fat thickness, and mature height [29].

The average SNP effect of missense, 3′ UTR and 5′ UTR variants on carcass traits was found to be greater than those of the other functional annotation categories, consistent with previous reports [12, 15]. For all traits studied, missense variants showed higher average genetic variance per SNP and greater squared allele substitution effects than synonymous variants. The stronger effects of missense variants on quantitative traits may be due to alternations in coding sequences and potentially affecting the function of the protein. Similar findings have been reported for carcass traits in Chinese Simmental beef cattle [52].

Understanding the genetic architecture of the traits is essential for improving the predictive ability of the models used in genetic evaluation programs. Using simulated data, Daetwyler et al. [7] demonstrated that BayesB provides higher accuracy than GBLUP when the number of QTLs is low, whereas GBLUP is more appropriate model for traits influenced by a large number of QTLs. The genetic architecture of quantitative traits can be explored through variant effect estimates obtained from GWAS. In this study, the distribution of allele substitution effects of the imputed WGS variants followed a thin-tailed normal distribution, where the majority of effects were extremely low and only a very small proportion of the SNPs had considerable effects. Overall, the mean of the effects was around zero due to that a big mass of SNPs with very small effects. Wang et al. [12] reported that hot carcass weight, rib eye area, lean meat yield traits are likely influenced by a few genes with large effects and many genes with small effects, while backfat thickness, and carcass marbling score are likely affected by a few genes with modest effects and many genes with small effects in multiple Canadian beef cattle breeds. The traits showed a very similar pattern in the distribution of SNP effects, with approximately 73, 95 and 99% of the marker effects falling within one, two and three standard deviations, respectively, from the overall mean of the SNP effects. These findings support the hypothesis that the marker effects and additive genetic variance for quantitative traits follow thin-tailed normal and scaled inverse chi-squared distributions, respectively. Similar findings have been previously reported for carcass merit [12], as well as feed efficiency and its component traits [15] in beef cattle.

In general, it seems that a few genes with modest effects plus a large number of genes with small effects contribute to the variation of all the studied traits, despite their small differences, and therefore the genetic architecture of the traits followed the assumption of quantitative genetics. Cole et al. [8] highlighted the importance of prior information for developing predictive models in dairy cattle breeding programs. The authors stated that, for example, for the traits like milk fat percentage the heavy-tailed model, corresponds to a model of mixture of many genes with small effects and a few genes with large effects, has higher prediction accuracy.

## Conclusions

The current study investigated the genomic regions associated with the carcass weight, marbling score, rib-eye area, and back fat thickness traits using 6,511,978 imputed whole genome sequence variants of 13,241 Angus beef cattle. Genome-wide association studies based on imputed WGS data identified several novel genomic regions associated with these traits, which were not detected using 50 K SNP chip array genotypes. In total, 13 significant signals were detected, among which two genomic regions on BTA7 and a region on BTA20 exhibited pleiotropic effects. On average, genic variants showed higher allele substitution effects compared to intergenic variants. Among the annotation functional categories, missense variants showed the largest SNP effect for CW, MS, and BFT, while 3′ UTR variants had higher effects for REA. The allele substitution effects of the imputed WGS variants followed a thin-tailed normal distribution, suggesting that the traits are controlled by a few variants with modest effects plus many variants with small effects, while the additive genetic variance explained by individual variants followed the scaled inverse chi-squared distribution. Our findings provide insight into the genetic architecture of carcass weight and meat quality traits in beef cattle, which can be applied to improve the accuracy of genomic prediction models in selective breeding programs.

## Supplementary Information

Additional file 1: Table S1. Chromosome length, number of SNPs and summary of marker interval for each chromosome.

Additional file 2: Table S2. Distribution of the SNPs over the functional annotation classes.

Additional file 3: Table S3. Average SNP effects, genetic variance of the SNP and the explained additive genetic variance of the trait for different functional annotation classes.

Additional file 4: Figure S1. Genetic architecture of carcass weight.

Additional file 5: Figure S2. Genetic architecture of marbling score.

Additional file 6: Figure S3. Genetic architecture of rib-eye area.

Additional file 7: Figure S4. Genetic architecture of back fat thickness.

Additional file 8: Figure S5. Distribution of estimated SNP effects for carcass weight (A), marbling score (B), rib-eye area (C) and back fat thickness (D).

Additional file 9: Figure S6. Distribution of significant SNPs over the autosomes.

Additional file 10: Table S4. Comparison of SNPs located within/close to genes vs. intergenic variants.

Additional file 11: Figure S7. Venn diagram of the significant SNP common in traits.

Additional file 12: Figure S8. Manhattan plot of GWAS for carcass weight (a), rib-eye area (b), marbling score (c), and back fat thickness (d), using IWGS variants (80–95 Mb on BTA7), without (left) and with (right) BTA7:90,819,463 as a fixed effect.

Additional file 13: Figure S9. Manhattan plot of GWAS for carcass weight (a), rib-eye area (b), marbling score (c), and back fat thickness (d), using IWGS variants (80–95 Mb on BTA7), without (left) and with (right) first 3 PCs as a covariate.

## Data Availability

The Whole Genome Sequence data of the samples used as reference population in this study are available from European Nucleotide Archive (ENA) at EMBL-EBI.
