# Supplementary material for: Genome-wide association studies and genetic architecture of carcass traits in Angus beef cattle using imputed whole-genome sequences data
Source: Genet Sel Evol. 2025 Jun 1;57:26. doi: 10.1186/s12711-025-00970-6 (PMC12128320; doi:10.1186/s12711-025-00970-6)
Supplement: Supplementary file 1 — Additional file 1: Table S1. Chromosome length, number of SNPs and summary of marker interval for each chromosome. [file 12711_2025_970_MOESM1_ESM.docx]

Supplementary Table 1. Marker density of bovine 50K SNP chip array and imputed whole genome sequence panel

| **BTA^1^** | **Length (Mb)** | **Bovine 50K SNP array** | | | |  | **Imputed whole genome sequence panel** | | | |  | **Density ratio^3^** |
| --- | --- | --- | --- | --- | --- | --- | --- | --- | --- | --- | --- | --- |
|  |  | **No. SNP^2^** | **Marker interval (bp)** | | |  | **No. SNP** | **Marker interval (bp)** | | |  |  |
|  |  |  | **Min** | **Max** | **Mean** |  |  | **Min** | **Max** | **Mean** |  |  |
| 1 | 157 | 3,204 | 131 | 497,531 | 49,097 |  | 451,494 | 1 | 240,215 | 350 |  | 140.92 |
| 2 | 136 | 2,618 | 32 | 597,667 | 51,950 |  | 352,103 | 1 | 354,137 | 382 |  | 134.49 |
| 3 | 121 | 2,402 | 1 | 799,833 | 50,256 |  | 312,673 | 1 | 326,021 | 387 |  | 130.17 |
| 4 | 120 | 2,407 | 4 | 335,688 | 49,709 |  | 312,754 | 1 | 286,441 | 383 |  | 129.94 |
| 5 | 120 | 2,117 | 1 | 727,739 | 56,653 |  | 271,721 | 1 | 590,038 | 442 |  | 128.35 |
| 6 | 117 | 2,860 | 1 | 541,894 | 41,076 |  | 368,198 | 1 | 440,976 | 320 |  | 128.74 |
| 7 | 110 | 2,623 | 1 | 637,072 | 41,802 |  | 282,397 | 1 | 290,617 | 392 |  | 107.66 |
| 8 | 111 | 2,391 | 1 | 355,146 | 46,549 |  | 276,607 | 1 | 444,895 | 409 |  | 115.69 |
| 9 | 104 | 2,054 | 1 | 663,781 | 50,738 |  | 246,313 | 1 | 305,590 | 424 |  | 119.92 |
| 10 | 103 | 2,294 | 2 | 3,049,437 | 44,947 |  | 273,550 | 1 | 1,181,758 | 378 |  | 119.25 |
| 11 | 107 | 2,193 | 1 | 539,424 | 48,643 |  | 268,698 | 1 | 217,101 | 396 |  | 122.53 |
| 12 | 87 | 1,657 | 111 | 1,068,983 | 52,536 |  | 229,000 | 1 | 982,989 | 380 |  | 138.20 |
| 13 | 83 | 1,707 | 5 | 726,077 | 48,589 |  | 195,198 | 1 | 646,635 | 427 |  | 114.35 |
| 14 | 82 | 1,679 | 1 | 575,373 | 48,829 |  | 214,646 | 1 | 261,799 | 383 |  | 127.84 |
| 15 | 84 | 1,620 | 666 | 913,993 | 51,989 |  | 215,280 | 1 | 365,559 | 394 |  | 132.89 |
| 16 | 81 | 1,647 | 1 | 475,821 | 48,938 |  | 200,102 | 1 | 454,530 | 405 |  | 121.49 |
| 17 | 73 | 1,613 | 1 | 623,632 | 45,185 |  | 222,995 | 1 | 172,473 | 328 |  | 138.25 |
| 18 | 65 | 1,298 | 4 | 786,167 | 50,270 |  | 149,284 | 1 | 233,339 | 437 |  | 115.01 |
| 19 | 63 | 1,317 | 14 | 333,198 | 47,821 |  | 146,122 | 1 | 280,917 | 434 |  | 110.95 |
| 20 | 71 | 1,518 | 674 | 504,949 | 47,007 |  | 200,773 | 1 | 278,968 | 357 |  | 132.26 |
| 21 | 69 | 1,411 | 2 | 922,571 | 49,188 |  | 159,833 | 1 | 654,446 | 434 |  | 113.28 |
| 22 | 61 | 1,240 | 7 | 315,020 | 48,816 |  | 152,216 | 1 | 279,710 | 398 |  | 122.75 |
| 23 | 52 | 1,078 | 1 | 572,482 | 48,501 |  | 166,250 | 1 | 142,342 | 316 |  | 154.22 |
| 24 | 62 | 1,218 | 95 | 427,626 | 50,977 |  | 172,528 | 1 | 136,712 | 361 |  | 141.65 |
| 25 | 42 | 948 | 1 | 234,159 | 44,557 |  | 125,939 | 1 | 68,926 | 336 |  | 132.85 |
| 26 | 51 | 1,029 | 281 | 519,638 | 49,906 |  | 134,892 | 1 | 253,127 | 385 |  | 131.09 |
| 27 | 44 | 999 | 4 | 436,605 | 44,319 |  | 130,372 | 1 | 133,281 | 350 |  | 130.50 |
| 28 | 45 | 870 | 23 | 360,814 | 52,220 |  | 132,345 | 1 | 460,326 | 347 |  | 152.12 |
| 29 | 50 | 997 | 127 | 824,889 | 50,534 |  | 147,695 | 1 | 412,660 | 345 |  | 148.14 |

1. BTA: *Bos taurus* autosomes; 2. No. SNP: Number of SNPs; 3. Interval ratio: Ratio of average marker interval (bp) in 50K chip array to WGS panel; 4. Density ratio: Ratio of average marker density (no. SNPs per Mb genome) in WGS panel to 50K chip array
