# Supplementary material for: Genome-wide association studies and genetic architecture of carcass traits in Angus beef cattle using imputed whole-genome sequences data
Source: Genet Sel Evol. 2025 Jun 1;57:26. doi: 10.1186/s12711-025-00970-6 (PMC12128320; doi:10.1186/s12711-025-00970-6)
Supplement: Supplementary file 2 — Additional file 2: Table S2. Distribution of the SNPs over the functional annotation classes. [file 12711_2025_970_MOESM2_ESM.docx]

Supplementary Table 2. Distribution of the SNPs over the functional annotations classes

| Functional class^1^ | No. SNPs^2^ | Class^3^ | Classification of SNP functions^4^ | No. SNPs^5^ | %^6^ | MAF^7^ ± SE |
| --- | --- | --- | --- | --- | --- | --- |
| Intergenic region | 3,840,411 | 1 | Intergenic region | 3,840,411 | 58.98 | 0.238±0.0001 |
| Downstream gene | 274,865 | 2 | Downstream | 274,865 | 4.22 | 0.234±0.0003 |
| Upstream gene | 288,857 | 3 | Upstream | 288,857 | 4.44 | 0.235±0.0003 |
| Synonymous | 18,785 | 4 | Synonymous | 22,284 | 0.34 | 0.236±0.0009 |
| Non-coding exon | 3,499 | 4 |  |  |  |  |
| Intron | 2,052,343 | 5 | Intron | 2,052,343 | 31.52 | 0.237±0.0001 |
| Missense | 13,140 | 6 | Missense | 13,140 | 0.20 | 0.228±0.0012 |
| 3’ UTR | 11,720 | 7 | 3’ UTR | 11,720 | 0.18 | 0.231±0.0013 |
| 5’ UTR | 2,878 | 8 | 5’ UTR | 2,878 | 0.04 | 0.231±0.0026 |
| Splice acceptor | 34 | 9 | Other regulatory region | 5,480 | 0.08 | 0.238±0.0019 |
| Splice donor | 634 | 9 |  |  |  |  |
| Splice region | 4,651 | 9 |  |  |  |  |
| Start lost | 34 | 9 |  |  |  |  |
| Stop lost | 111 | 9 |  |  |  |  |
| Stop retained | 16 | 9 |  |  |  |  |

1. Functional class annotation for variants obtained originally provide by Ensemble; 2. Number of SNPs in different functional original annotations; 3. The class number of functional classes grouped in this study; 4. The functional classes’ annotation by grouping the original annotation classes; 5. Number of SNPs in the classified functional annotations; 6. Percentage of SNPs belonging to the functional classes; 7. Minor allele frequency
