# Supplementary material for: Genome-wide association studies and genetic architecture of carcass traits in Angus beef cattle using imputed whole-genome sequences data
Source: Genet Sel Evol. 2025 Jun 1;57:26. doi: 10.1186/s12711-025-00970-6 (PMC12128320; doi:10.1186/s12711-025-00970-6)
Supplement: Supplementary file 3 — Additional file 3: Table S3. Average SNP effects, genetic variance of the SNP and the explained additive genetic variance of the trait for different functional annotation classes. [file 12711_2025_970_MOESM3_ESM.docx]

Supplementary Table 3. Average SNP effects, genetic variance of the SNP and the explained additive genetic variance of the trait for different functional annotation classes

| **Trait** | **Functions classes** | **Group. No** | **No. SNP** | **𝛽^2^ ± SE** | **Ratio_𝛽^2^** | **Vg ± SE** | **No. SNP_O** | **Vg_O ± SE** | **Ratio_Vg** | **EVG ± SE** |
| --- | --- | --- | --- | --- | --- | --- | --- | --- | --- | --- |
| CW | Intergenic region | 1 | 3,840,411 | 0.517 ± 0.001 | 0.984 | 1.141E-1 ± 9.280E-5 | 2671567 | 1.213E-1 ± 1.149e-4 | 0.941 | 4.64E-2 ± 3.77E-5 |
|  | Downstream gene | 2 | 274,865 | 0.564 ± 0.003 | 1.073 | 1.276E-1 ± 3.918E-4 | 6237113 | 1.166E-1 ± 7.342E-5 | 1.095 | 5.18E-2 ± 1.59E-4 |
|  | Upstream gene | 3 | 288,857 | 0.543 ± 0.002 | 1.033 | 1.238E-1 ± 3.476E-4 | 6223121 | 1.168E-1 ± 7.385E-5 | 1.060 | 5.03E-2 ± 1.41E-4 |
|  | Synonymous | 4 | 22,284 | 0.556 ± 0.008 | 1.059 | 1.257E-1 ± 1.272E-3 | 6489694 | 1.170E-1 ± 7.236E-5 | 1.074 | 5.10E-2 ± 5.17E-4 |
|  | Intron | 5 | 2,052,343 | 0.533 ± 0.001 | 1.014 | 1.199E-1 ± 1.291E-4 | 4459635 | 1.158E-1 ± 8.718E-5 | 1.036 | 4.87E-2 ± 5.24E-5 |
|  | Missense | 6 | 13,140 | 0.606 ± 0.011 | 1.152 | 1.354E-1 ± 1.912E-3 | 6498838 | 1.170E-1 ± 7.229E-5 | 1.157 | 5.50E-2 ± 7.76E-4 |
|  | 3 UTR | 7 | 11,720 | 0.553 ± 0.013 | 1.052 | 1.264E-1 ± 2.144E-3 | 6500258 | 1.171E-1 ± 7.227E-5 | 1.080 | 5.13E-2 ± 8.71E-4 |
|  | 5 UTR | 8 | 2,878 | 0.533 ± 0.021 | 1.014 | 1.197E-1 ± 3.216E-3 | 6509100 | 1.171E-1 ± 7.226E-5 | 1.023 | 4.86E-2 ± 1.31E-3 |
|  | Other regulatory region | 9 | 5,480 | 0.540 ± 0.014 | 1.028 | 1.294E-1 ± 2.648E-3 | 6506498 | 1.171E-1 ± 7.227E-5 | 1.106 | 5.26E-2 ± 1.08E-3 |
| FAT | Intergenic region | 1 | 3,840,411 | 2.824e-5 ± 3.017e-8 | 0.981 | 6.386E-6 ± 4.837E-9 | 2671567 | 6.502E-6 ± 5.845E-9 | 0.982 | 6.60E-2 ± 5.00E-5 |
|  | Downstream gene | 2 | 274,865 | 2.975e-5 ± 1.204e-7 | 1.033 | 6.554E-6 ± 1.846E-8 | 6237113 | 6.428E-6 ± 3.805E-9 | 1.020 | 6.78E-2 ± 1.91E-4 |
|  | Upstream gene | 3 | 288,857 | 2.971e-5 ± 1.191e-7 | 1.032 | 6.550E-6 ± 1.799E-8 | 6223121 | 6.428E-6 ± 3.809E-9 | 1.019 | 6.77E-2 ± 1.86E-4 |
|  | Synonymous | 4 | 22,284 | 2.916e-5 ± 4.349e-7 | 1.012 | 6.419E-6 ± 6.152E-8 | 6489694 | 6.433E-6 ± 3.733E-9 | 0.998 | 6.64E-2 ± 6.36E-4 |
|  | Intron | 5 | 2,052,343 | 2.958e-5 ± 4.490e-8 | 1.027 | 6.490E-6 ± 6.648E-9 | 4459635 | 6.407E-6 ± 4.500E-9 | 1.013 | 6.71E-2 ± 6.87E-5 |
|  | Missense | 6 | 13,140 | 3.096e-5 ± 6.083e-7 | 1.075 | 6.370E-6 ± 8.268E-8 | 6498838 | 6.433E-6 ± 3.730E-9 | 0.990 | 6.59E-2 ± 8.55E-4 |
|  | 3 UTR | 7 | 11,720 | 2.824e-5 ± 5.248e-7 | 0.981 | 6.312E-6 ± 8.641E-8 | 6500258 | 6.433E-6 ± 3.730E-9 | 0.981 | 6.53E-2 ± 8.93E-4 |
|  | 5 UTR | 8 | 2,878 | 3.096e-5 ± 1.355e-6 | 1.075 | 6.785E-6 ± 1.972E-7 | 6509100 | 6.433E-6 ± 3.727E-9 | 1.055 | 7.01E-2 ± 2.04E-3 |
|  | Other regulatory region | 9 | 5,480 | 2.939e-5 ± 9.050e-7 | 1.021 | 6.546E-6 ± 1.286E-7 | 6506498 | 6.433E-6 ± 3.728E-9 | 1.017 | 6.77E-2 ± 1.33E-3 |
|  | Intergenic region | 1 | 3,840,411 | 8.076e-4 ± 8.335e-7 | 0.970 | 1.864E-4 ± 1.426E-7 | 2671567 | 2.008E-4 ± 1.864E-7 | 0.928 | 3.41E-2 ± 2.61E-5 |
| MS | Downstream gene | 2 | 274,865 | 9.245e-4 ± 3.594e-6 | 1.110 | 2.100E-4 ± 6.125E-7 | 6237113 | 1.915E-4 ± 1.156E-7 | 1.097 | 3.84E-2 ± 1.12E-4 |
|  | Upstream gene | 3 | 288,857 | 9.279e-4 ± 3.519e-6 | 1.114 | 2.107E-4 ± 5.907E-7 | 6223121 | 1.914E-4 ± 1.158E-7 | 1.100 | 3.85E-2 ± 1.08E-4 |
|  | Synonymous | 4 | 22,284 | 9.046e-4 ± 1.272e-5 | 1.086 | 2.090E-4 ± 2.228E-6 | 6489694 | 1.922E-4 ± 1.139E-7 | 1.087 | 3.82E-2 ± 4.07E-4 |
|  | Intron | 5 | 2,052,343 | 8.518e-4 ± 1.192e-6 | 1.023 | 1.980E-4 ± 2.094E-7 | 4459635 | 1.897E-4 ± 1.352E-7 | 1.044 | 3.62E-2 ± 3.83E-5 |
|  | Missense | 6 | 13,140 | 9.647e-4 ± 1.741e-5 | 1.159 | 2.112E-4 ± 2.805E-6 | 6498838 | 1.922E-4 ± 1.138E-7 | 1.099 | 3.86E-2 ± 5.13E-4 |
|  | 3 UTR | 7 | 11,720 | 8.635e-4 ± 1.537e-5 | 1.037 | 2.004E-4 ± 2.721E-6 | 6500258 | 1.923E-4 ± 1.138E-7 | 1.042 | 3.66E-2 ± 4.97E-4 |
|  | 5 UTR | 8 | 2,878 | 9.295e-4 ± 3.246e-5 | 1.116 | 2.103E-4 ± 6.308E-6 | 6509100 | 1.923E-4 ± 1.137E-7 | 1.094 | 3.84E-2 ± 1.15E-3 |
|  | Other regulatory region | 9 | 5,480 | 8.717e-4 ± 2.335e-5 | 1.047 | 2.059E-4 ± 4.400E-6 | 6506498 | 1.923E-4 ± 1.138E-7 | 1.071 | 3.76E-2 ± 8.04E-4 |
| REA | Intergenic region | 1 | 3,840,411 | 8.554e-4 ± 9.428e-7 | 0.998 | 1.943E-4 ± 1.579E-7 | 2671567 | 1.966E-4 ± 1.863E-7 | 0.988 | 4.50E-2 ± 3.66E-5 |
|  | Downstream gene | 2 | 274,865 | 8.69e-4 ± 3.57e-6 | 1.015 | 1.980E-4 ± 5.761E-7 | 6237113 | 1.952E-4 ± 1.232E-7 | 1.014 | 4.59E-2 ± 1.33E-4 |
|  | Upstream gene | 3 | 288,857 | 8.49e-4 ± 3.39e-6 | 0.991 | 1.955E-4 ± 5.436E-7 | 6223121 | 1.953E-4 ± 1.235E-7 | 1.001 | 4.53E-2 ± 1.26E-4 |
|  | Synonymous | 4 | 22,284 | 8.44e-4 ± 1.19e-5 | 0.985 | 1.968E-4 ± 1.948E-6 | 6489694 | 1.953E-4 ± 1.207E-7 | 1.008 | 4.56E-2 ± 4.51E-4 |
|  | Intron | 5 | 2,052,343 | 8.58e-4 ± 1.29e-6 | 1.002 | 1.965E-4 ± 2.141E-7 | 4459635 | 1.947E-4 ± 1.458E-7 | 1.010 | 4.55E-2 ± 4.96E-5 |
|  | Missense | 6 | 13,140 | 8.93e-4 ± 1.58e-5 | 1.042 | 2.001E-4 ± 2.627E-6 | 6498838 | 1.953E-4 ± 1.206E-7 | 1.025 | 4.64E-2 ± 6.09E-4 |
|  | 3 UTR | 7 | 11,720 | 9.11e-4 ± 1.94e-5 | 1.063 | 2.013E-4 ± 2.758E-6 | 6500258 | 1.953E-4 ± 1.206E-7 | 1.031 | 4.67E-2 ± 6.39E-4 |
|  | 5 UTR | 8 | 2,878 | 8.67e-4 ± 3.23e-5 | 1.012 | 2.025E-4 ± 5.690E-6 | 6509100 | 1.953E-4 ± 1.205E-7 | 1.037 | 4.69E-2 ± 1.32E-3 |
|  | Other regulatory region | 9 | 5,480 | 8.92e-4 ± 2.97e-5 | 1.042 | 2.010E-4 ± 4.250E-6 | 6506498 | 1.953E-4 ± 1.205E-7 | 1.030 | 4.66E-2 ± 9.85E-4 |
