# Supplementary figures and images for: Genome-wide association studies and genetic architecture of carcass traits in Angus beef cattle using imputed whole-genome sequences data
Source: Genet Sel Evol. 2025 Jun 1;57:26. doi: 10.1186/s12711-025-00970-6 (PMC12128320; doi:10.1186/s12711-025-00970-6)

# Carcass Weight

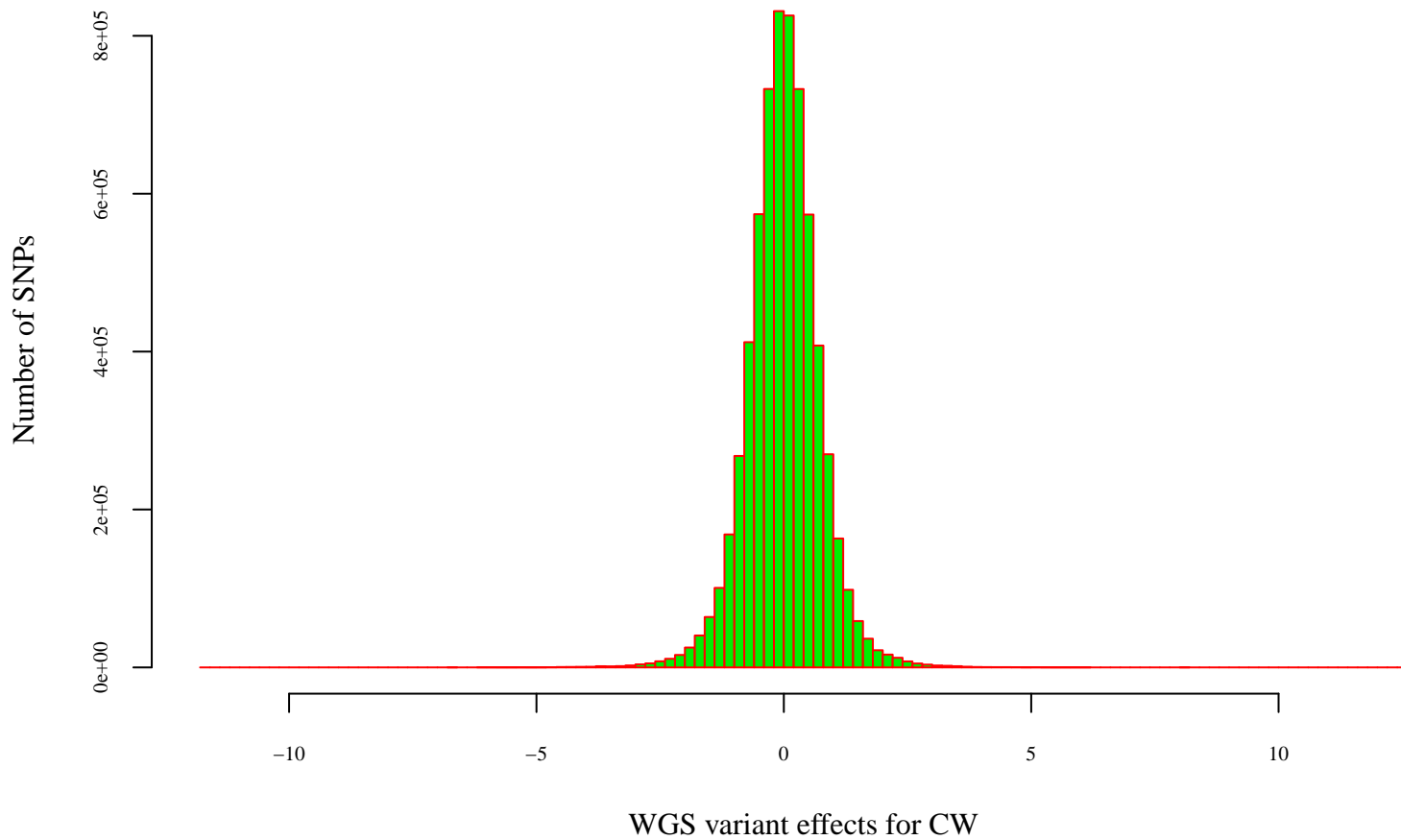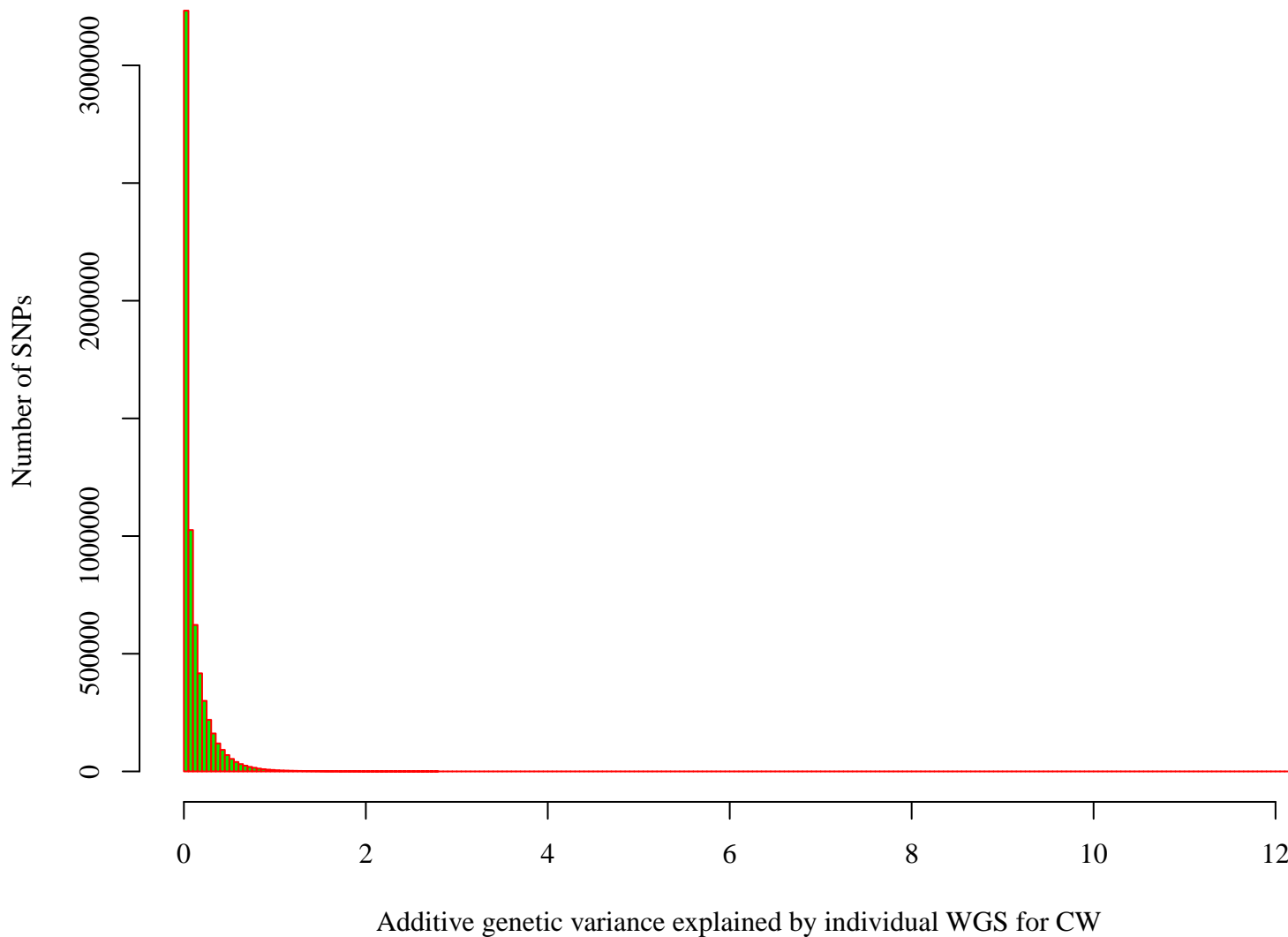

Supplement: Supplementary file 4 — Additional file 4: Figure S1. Genetic architecture of carcass weight. [file 12711_2025_970_MOESM4_ESM.pdf]

## Marbling Score

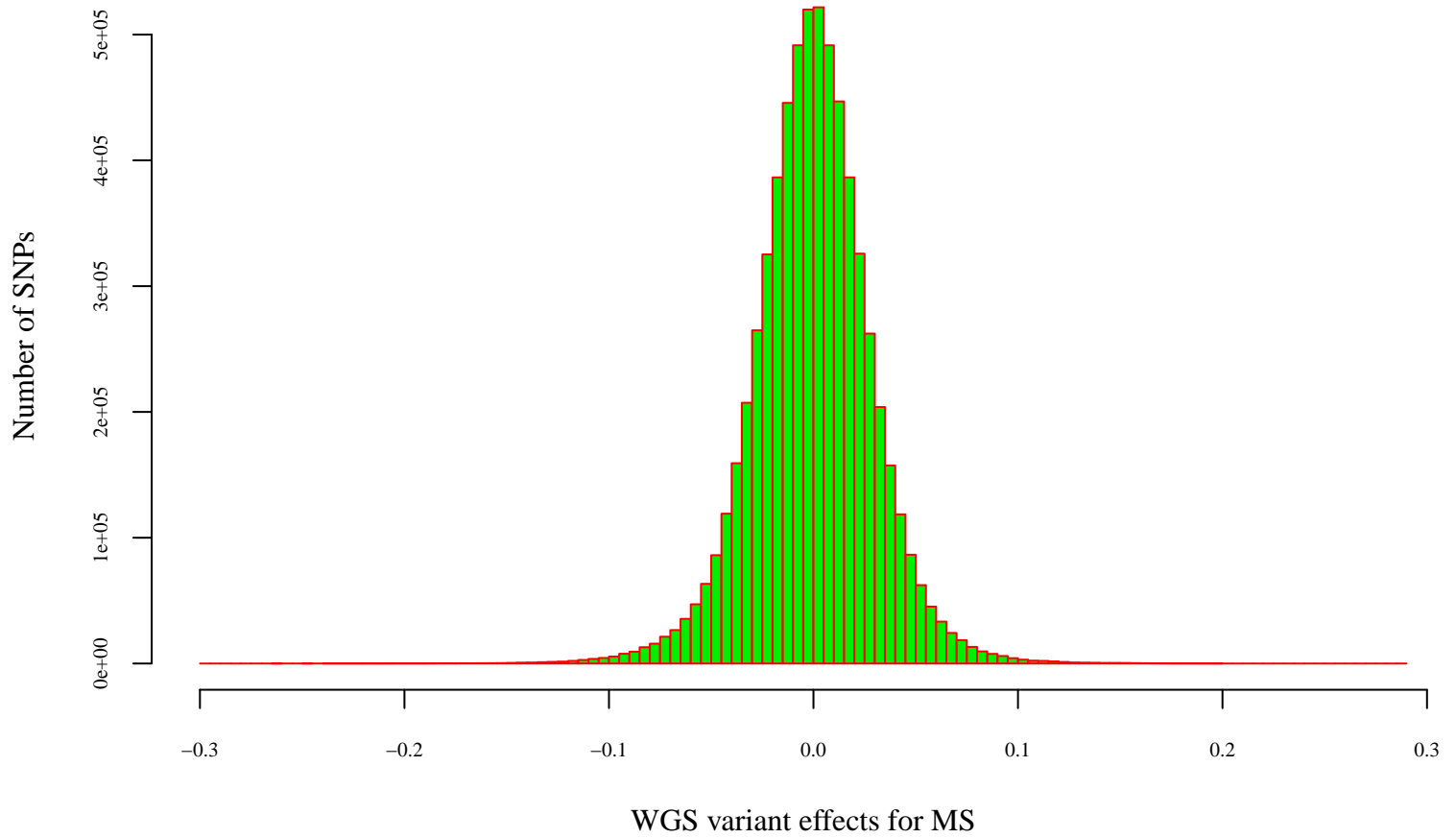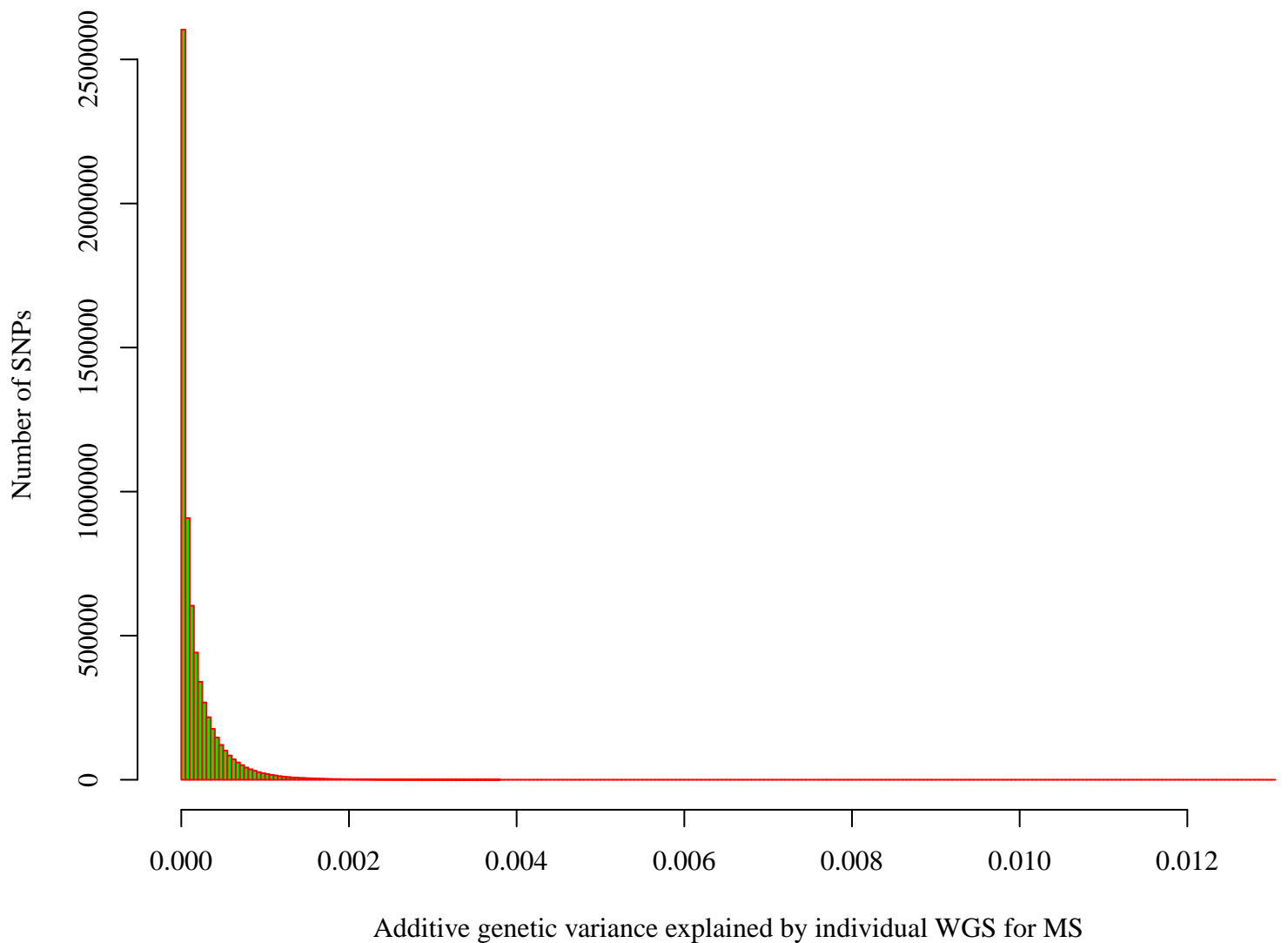

Supplement: Supplementary file 5 — Additional file 5: Figure S2. Genetic architecture of marbling score. [file 12711_2025_970_MOESM5_ESM.pdf]

## Rib Eye Area

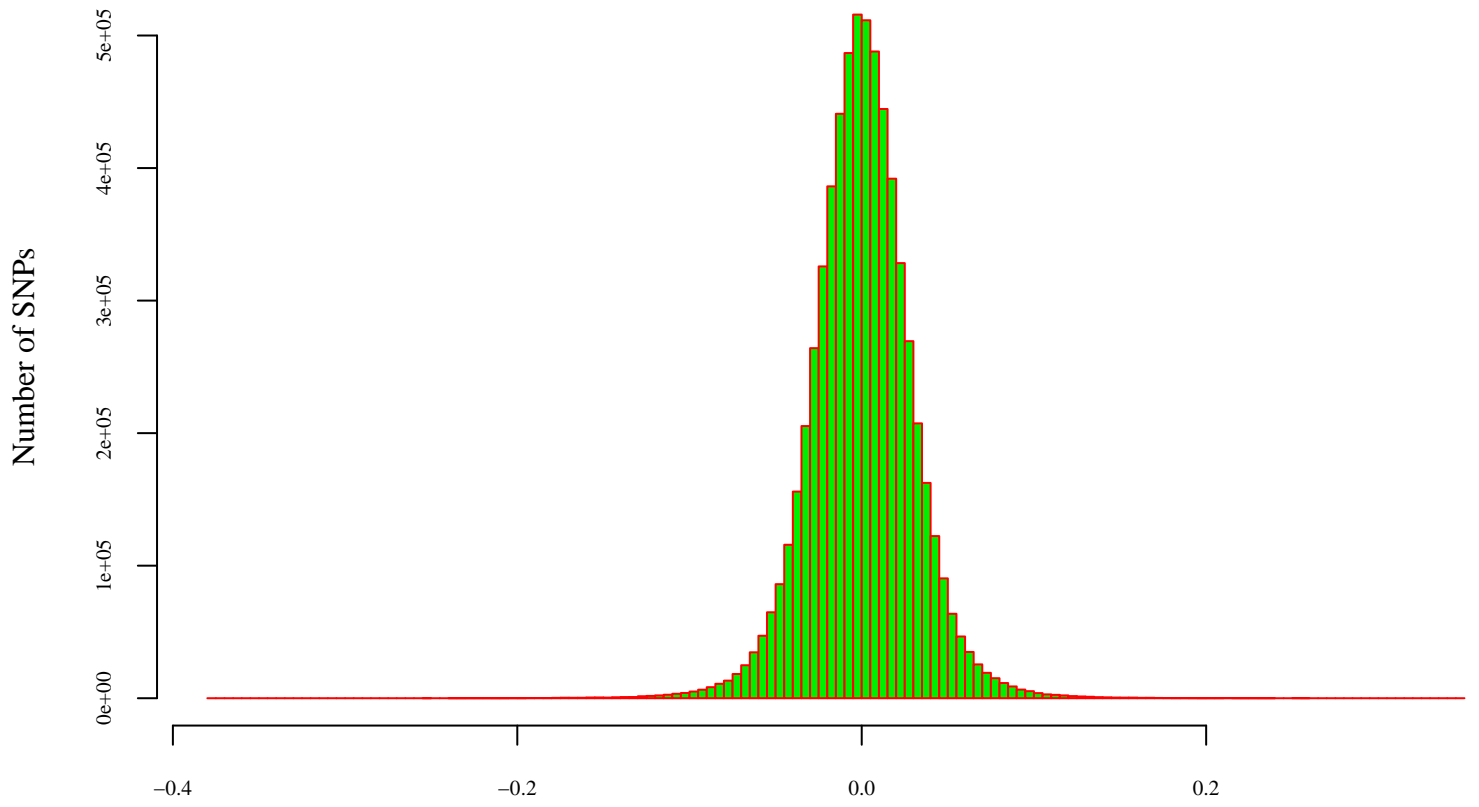

## WGS variant effects for REA

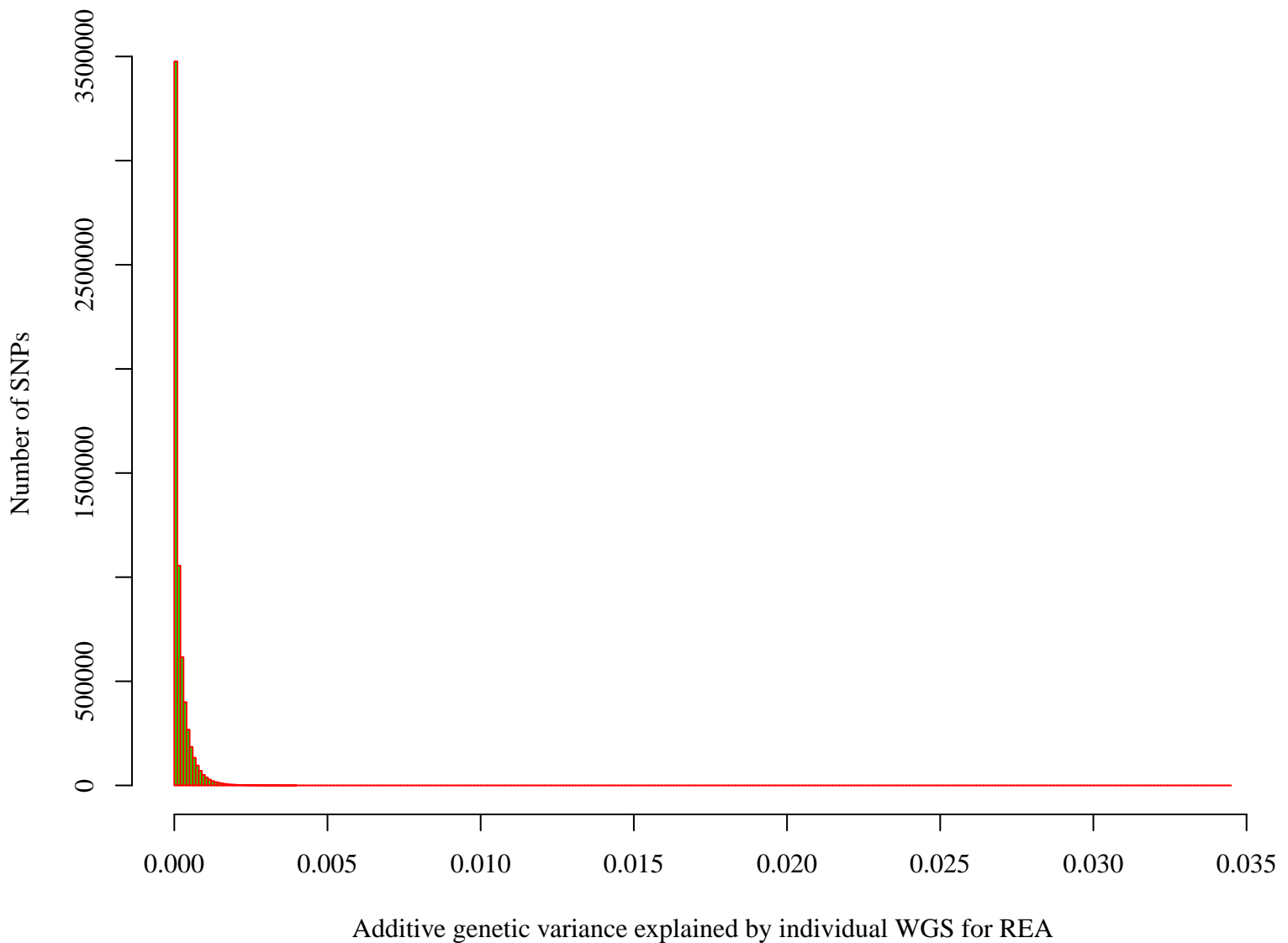

Supplement: Supplementary file 6 — Additional file 6: Figure S3. Genetic architecture of rib-eye area. [file 12711_2025_970_MOESM6_ESM.pdf]

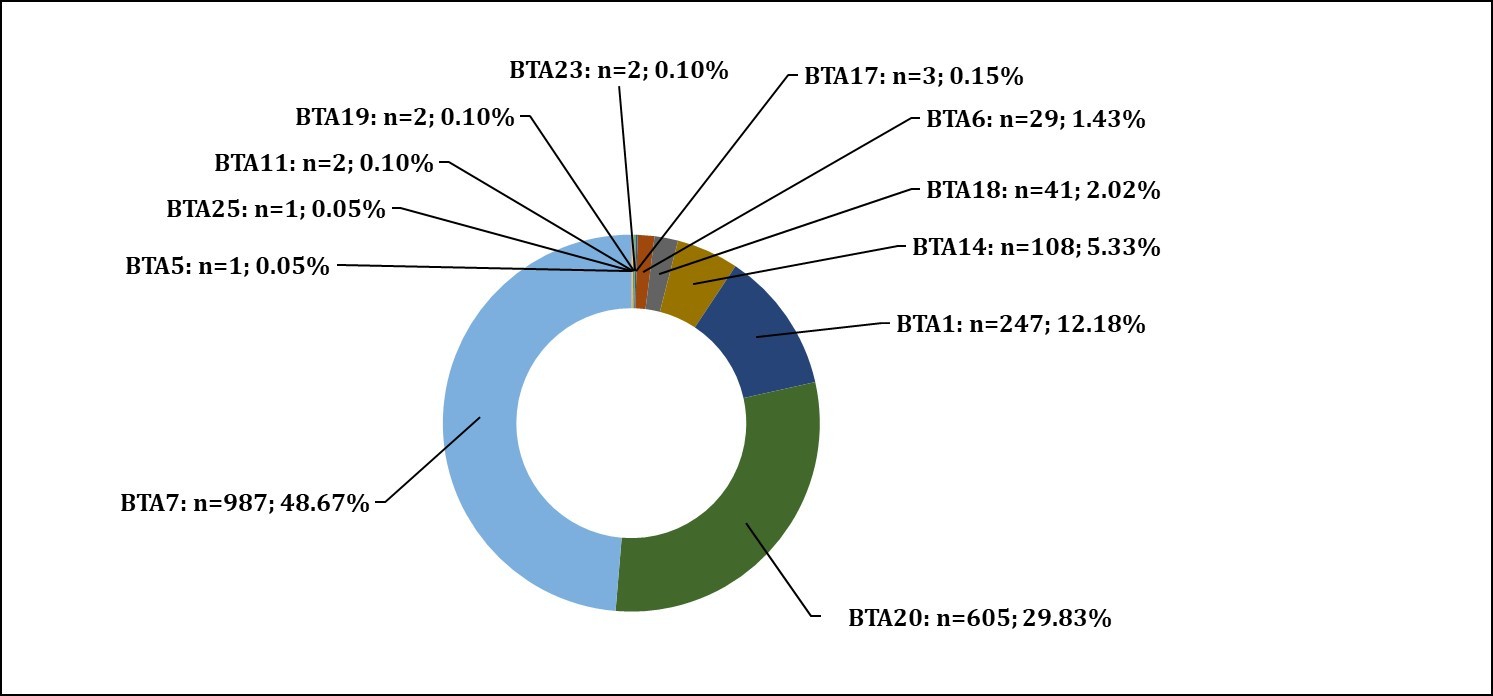


**Figure S6:** Distribution of significant SNPs over the autosomes.

Supplement: Supplementary file 9 — Additional file 9: Figure S6. Distribution of significant SNPs over the autosomes. [file 12711_2025_970_MOESM9_ESM.docx]

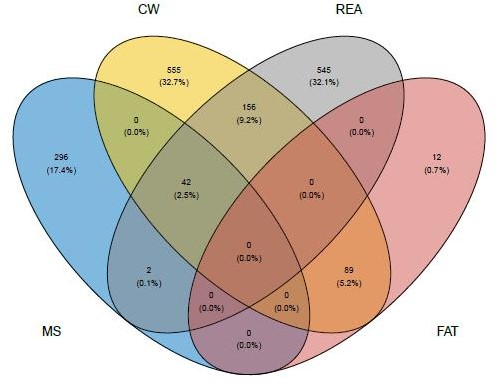


**Figure S7:** Venn diagram of the significant SNP common in traits.

Supplement: Supplementary file 11 — Additional file 11: Figure S7. Venn diagram of the significant SNP common in traits. [file 12711_2025_970_MOESM11_ESM.docx]
