# Supplementary material for: Genome-wide association studies and genetic architecture of carcass traits in Angus beef cattle using imputed whole-genome sequences data
Source: Genet Sel Evol. 2025 Jun 1;57:26. doi: 10.1186/s12711-025-00970-6 (PMC12128320; doi:10.1186/s12711-025-00970-6)
Supplement: Supplementary file 7 — Additional file 7: Figure S4. Genetic architecture of back fat thickness. [file 12711_2025_970_MOESM7_ESM.pdf]

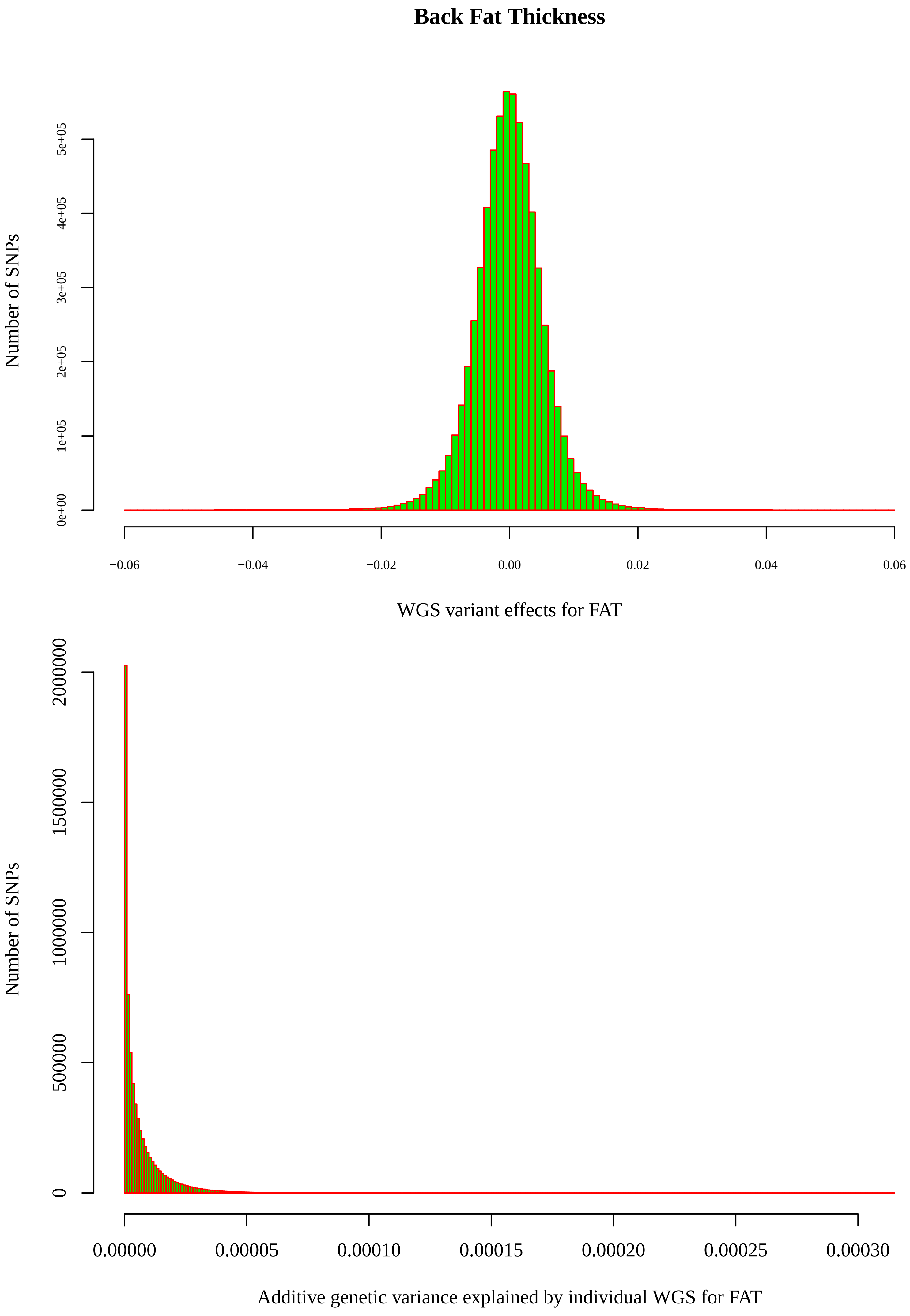

Back Fat Thickness
Number of SNPs
0e+00
1e+05
2e+05
3e+05
4e+05
5e+05
−0.06
−0.04
−0.02
0.00
0.02
0.04
0.06
WGS variant effects for FAT
Number of SNPs
0
500000
1000000
1500000
2000000
0.00000
0.00005
0.00010
0.00015
0.00020
0.00025
0.00030
Additive genetic variance explained by individual WGS for FAT
