# Supplementary material for: Genome-wide association studies and genetic architecture of carcass traits in Angus beef cattle using imputed whole-genome sequences data
Source: Genet Sel Evol. 2025 Jun 1;57:26. doi: 10.1186/s12711-025-00970-6 (PMC12128320; doi:10.1186/s12711-025-00970-6)
Supplement: Supplementary file 8 — Additional file 8: Figure S5. Distribution of estimated SNP effects for carcass weight (A), marbling score (B), rib-eye area (C) and back fat thickness (D). [file 12711_2025_970_MOESM8_ESM.docx]

| **A** |  | **B** |
| --- | --- | --- |
| 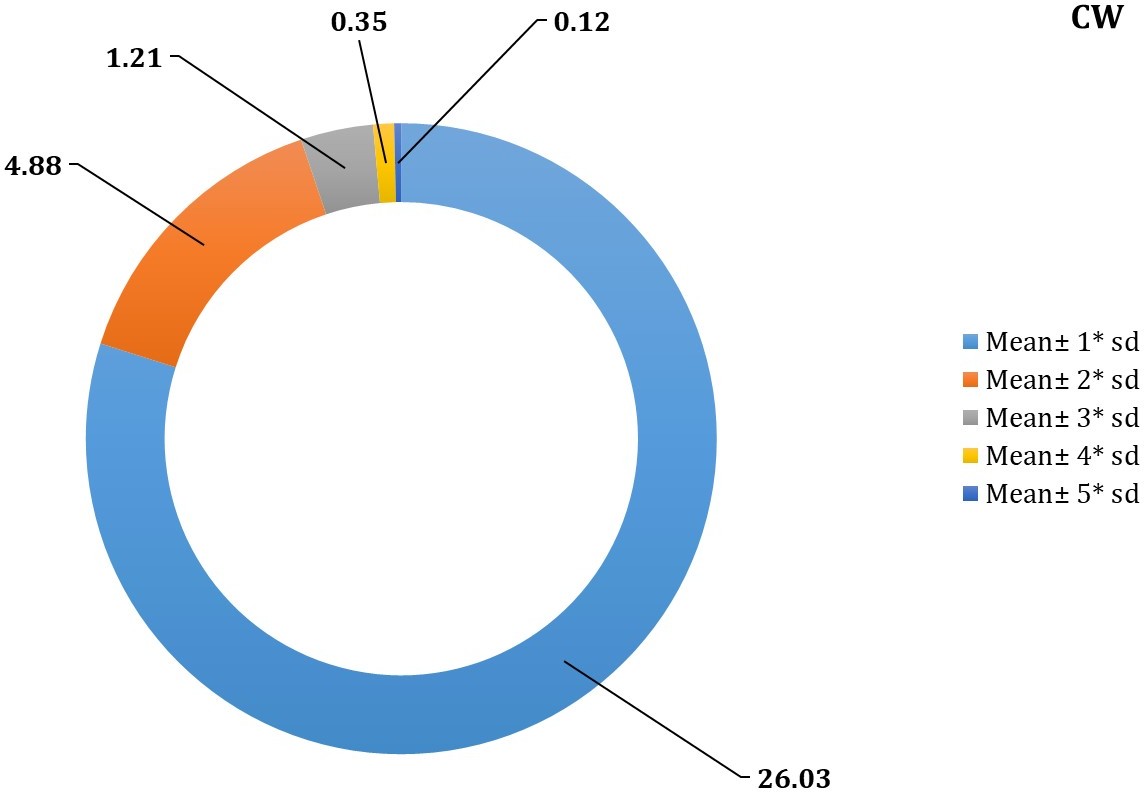 |  | 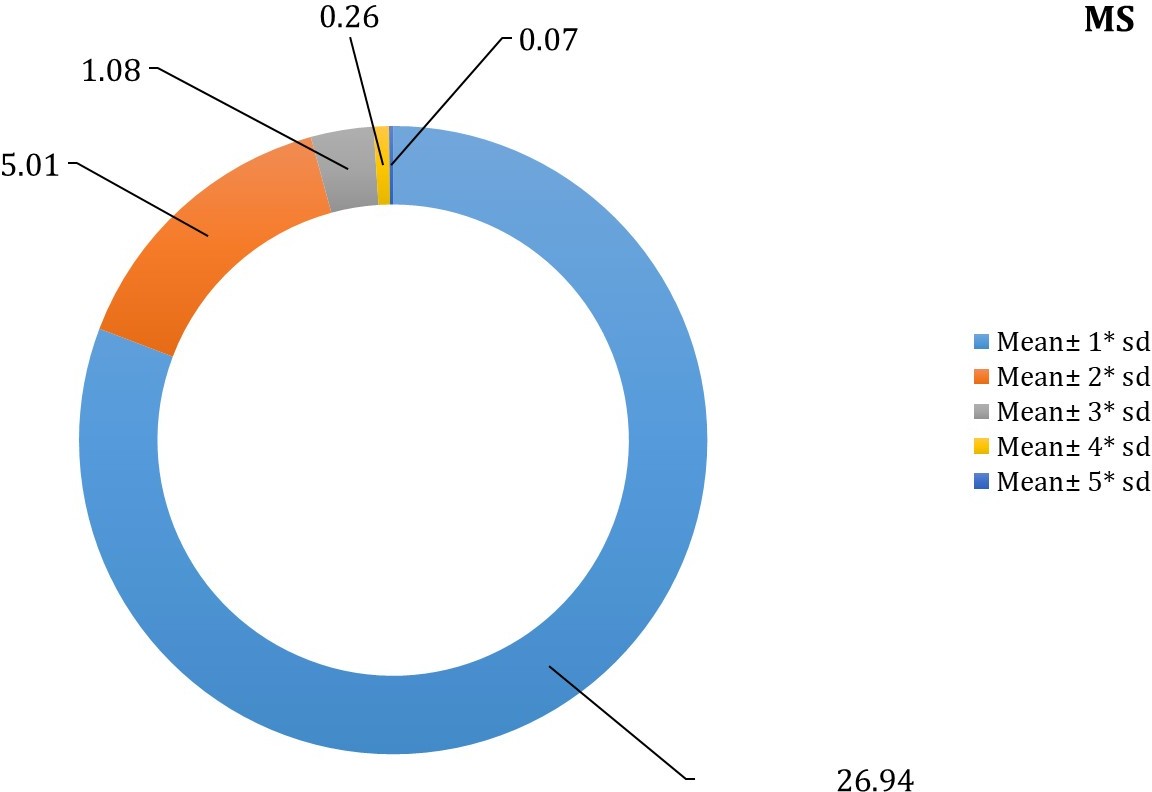 |
| **C** |  | **D** |
| 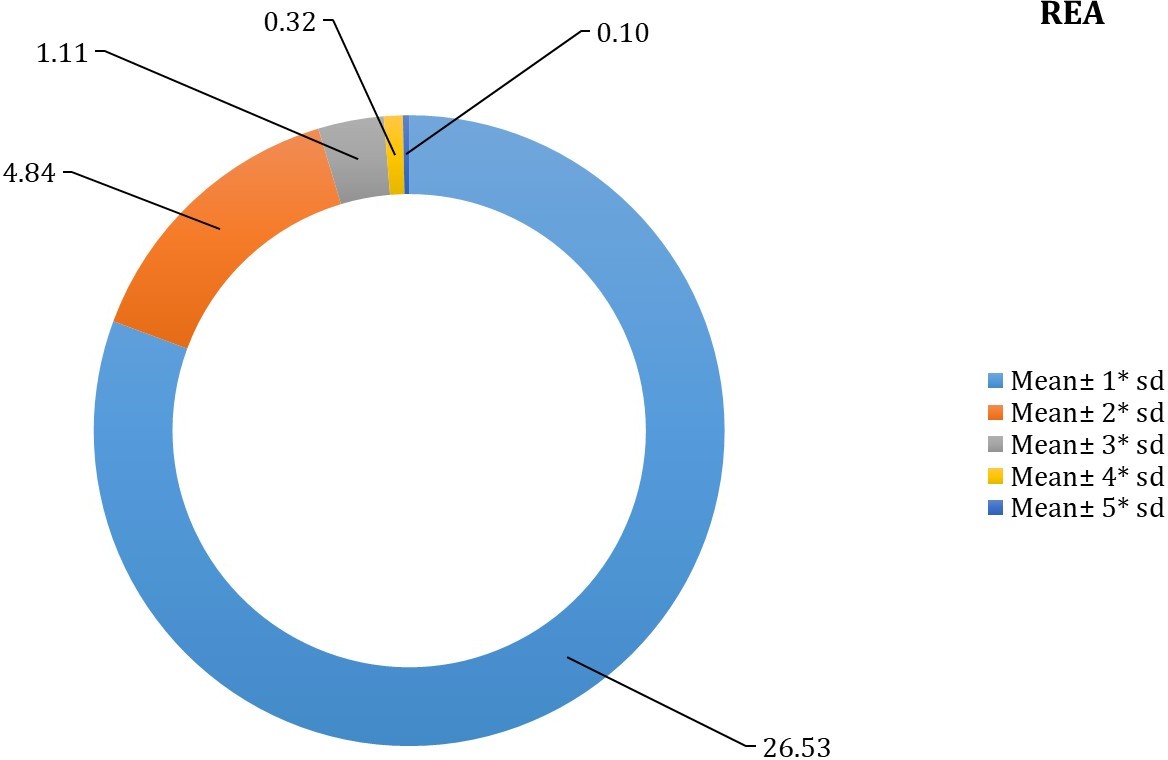 |  | 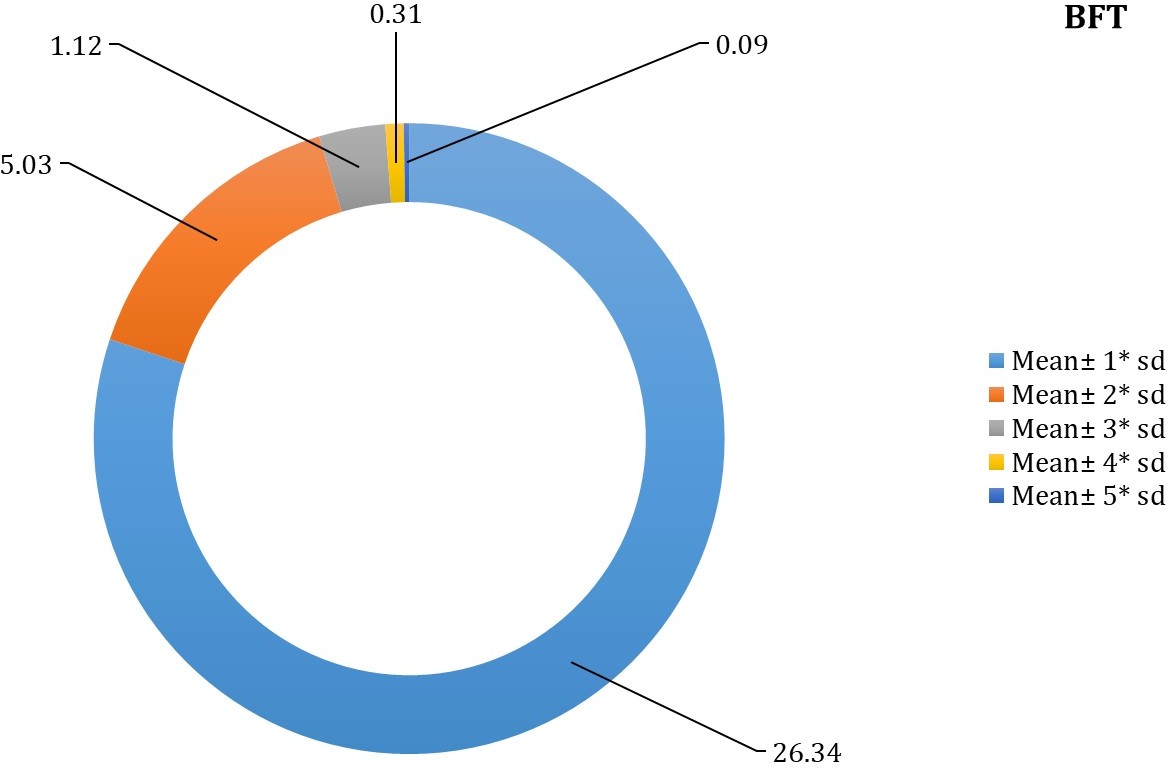 |

**Figure S5:** Distribution of estimated SNP effects for carcass weight (A), marbling score (B), rib-eye area (C) and back fat thickness (D)
