# Supplementary material for: Genome-wide association studies and genetic architecture of carcass traits in Angus beef cattle using imputed whole-genome sequences data
Source: Genet Sel Evol. 2025 Jun 1;57:26. doi: 10.1186/s12711-025-00970-6 (PMC12128320; doi:10.1186/s12711-025-00970-6)
Supplement: Supplementary file 10 — Additional file 10: Table S4. Comparison of SNPs located within/close to genes vs. intergenic variants. [file 12711_2025_970_MOESM10_ESM.docx]

Supplementary Table S4. Comparison of SNPs located within/close to genes vs. intergenic variants

| Parameter | CW | |  | MS | |  | REA | |
| --- | --- | --- | --- | --- | --- | --- | --- | --- |
|  | Mapped  (n=655) | Not mapped  (n=187) |  | Mapped  (n=322) | Not mapped  (n=18) |  | Mapped  (n=371) | Not mapped  (n=374) |
| MAF | 0.300 | 0.333 |  | 0.261 | 0.311 |  | 0.318 | 0.304 |
| SNP effect | 22.2218 | 10.6151 |  | 0.0193 | 0.0149 |  | 0.0238 | 0.0226 |
| Vg | 4.997 | 3.909 |  | 0.007 | 0.006 |  | 0.010 | 0.008 |
| EVG | 2.029 | 1.588 |  | 1.231 | 1.138 |  | 2.280 | 1.928 |
