# Supplementary material for: Genome-wide association studies and genetic architecture of carcass traits in Angus beef cattle using imputed whole-genome sequences data
Source: Genet Sel Evol. 2025 Jun 1;57:26. doi: 10.1186/s12711-025-00970-6 (PMC12128320; doi:10.1186/s12711-025-00970-6)
Supplement: Supplementary file 12 — Additional file 12: Figure S8. Manhattan plot of GWAS for carcass weight (a), rib-eye area (b), marbling score (c), and back fat thickness (d), using IWGS variants (80–95 Mb on BTA7), without (left) and with (right) BTA7:90,819,463 as a fixed effect. [file 12711_2025_970_MOESM12_ESM.docx]

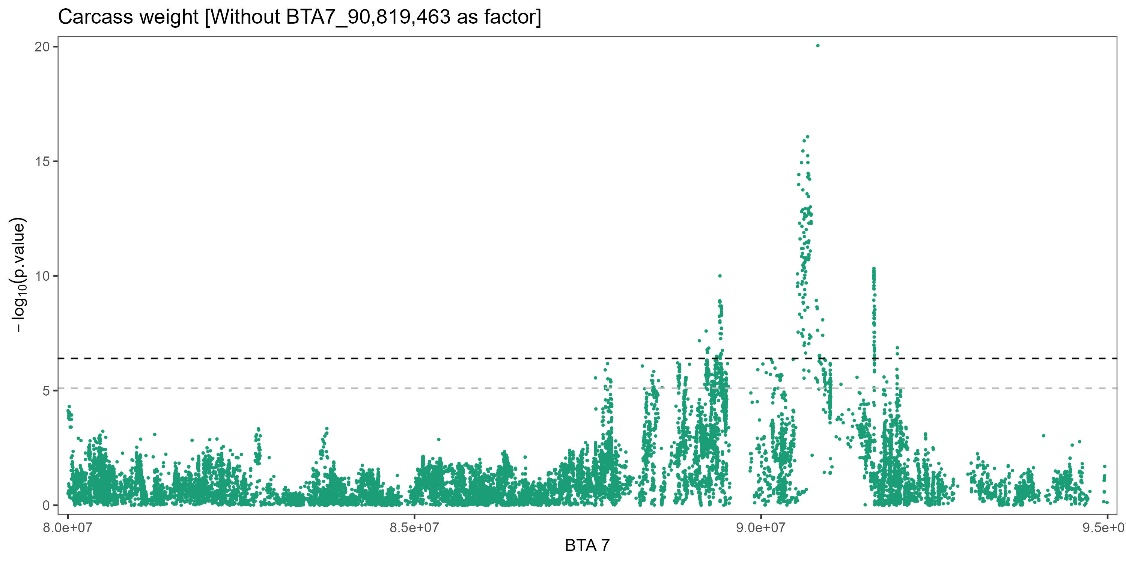

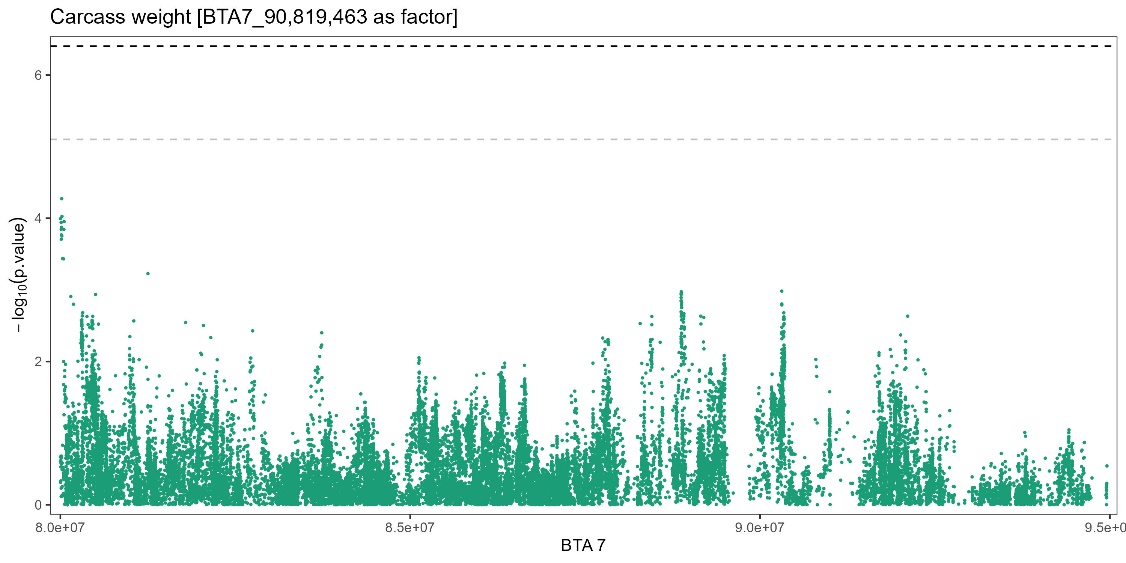


(a) Manhattan plot of GWAS for carcass weight using IWGS variants (80–95 Mb on BTA7), without (left) and with (right) BTA7:90,819,463 as a fixed effect.


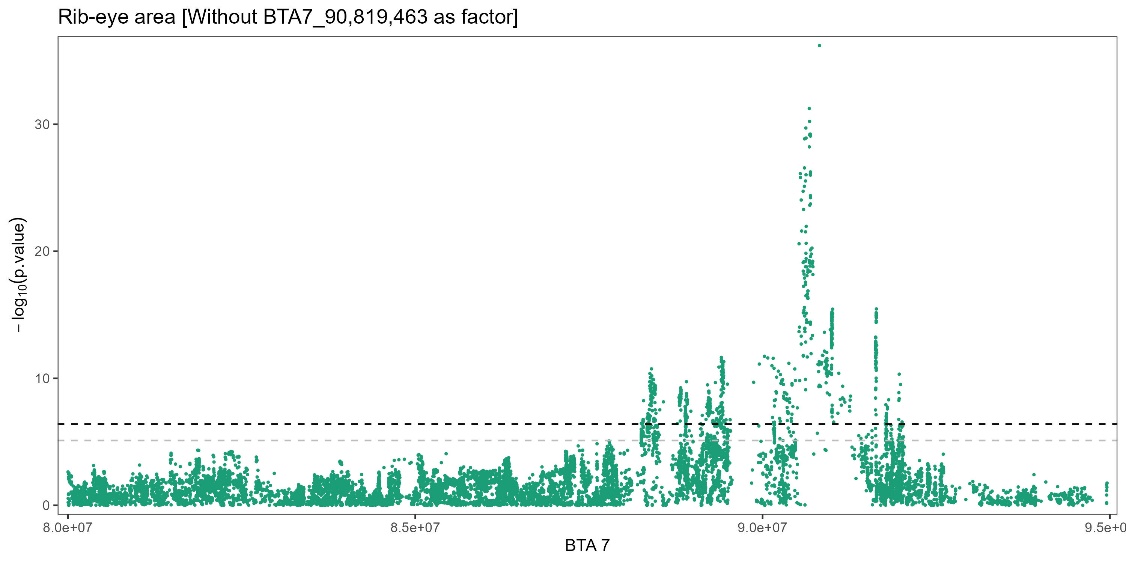

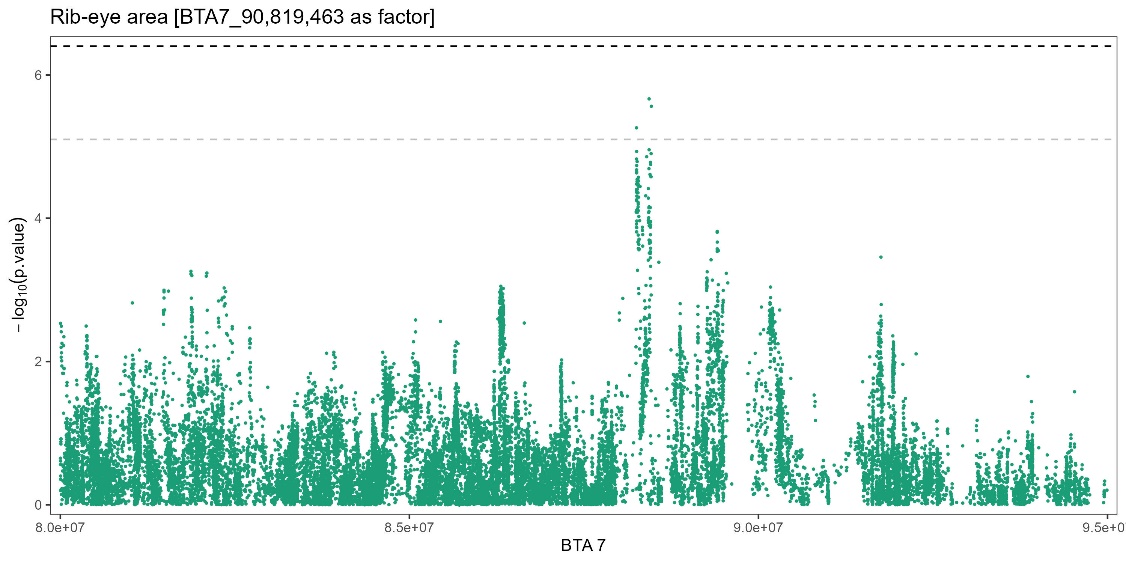


(b)Manhattan plot of GWAS for rib-eye area using IWGS variants (80–95 Mb on BTA7), without (left) and with (right) BTA7:90,819,463 as a fixed effect.


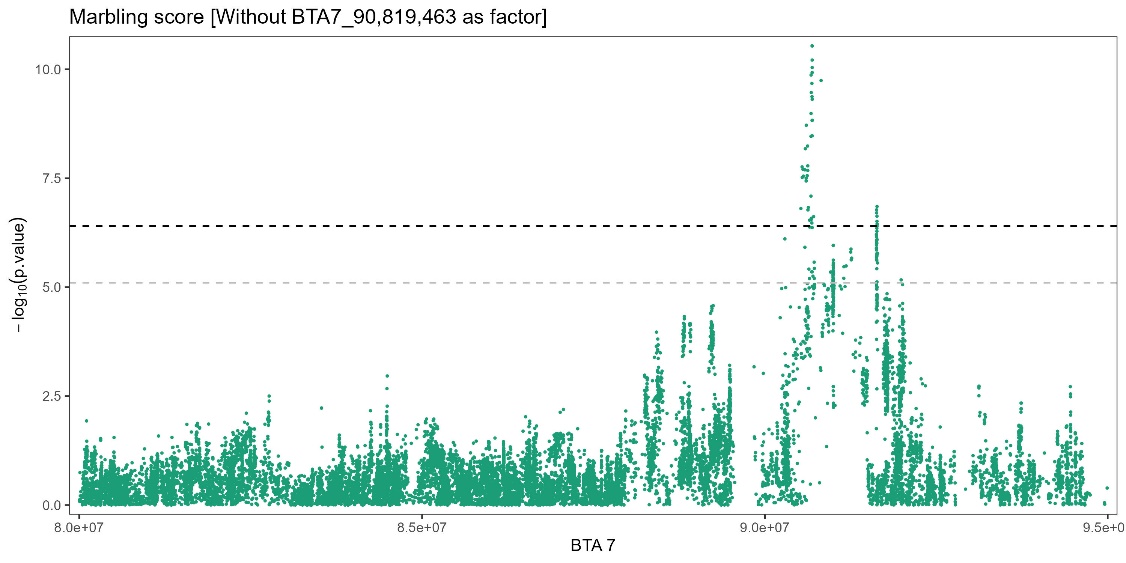

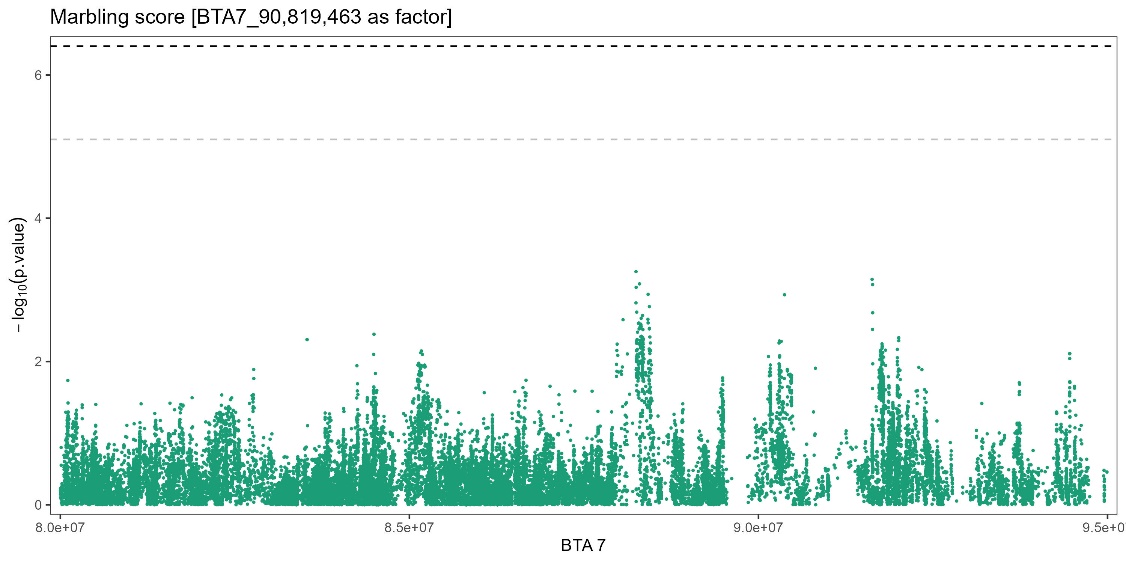


(c) Manhattan plot of GWAS for marbling score using IWGS variants (80–95 Mb on BTA7), without (left) and with (right) BTA7:90,819,463 as a fixed effect.


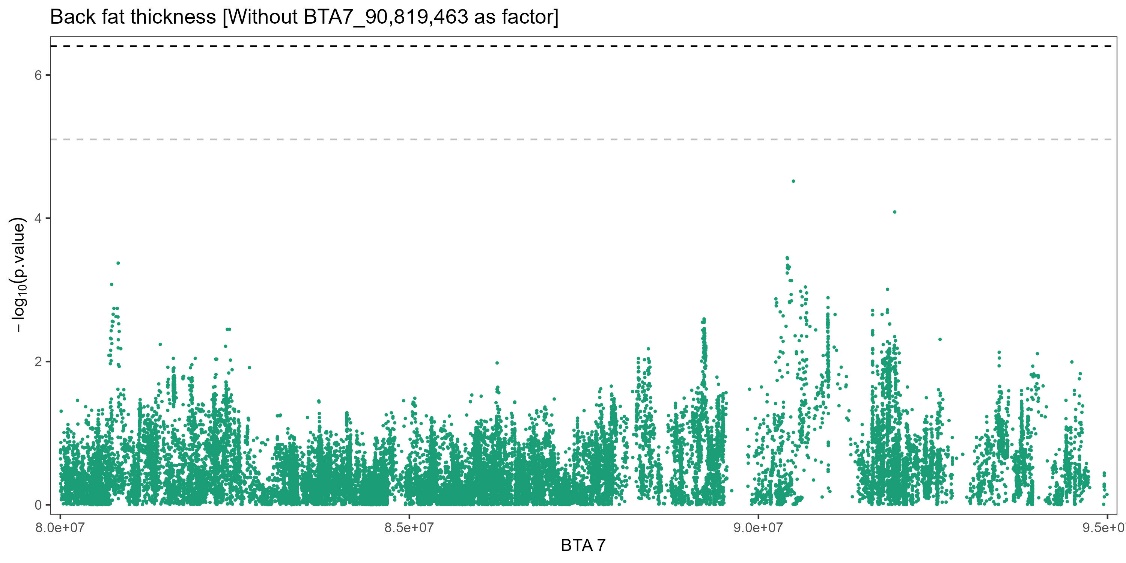

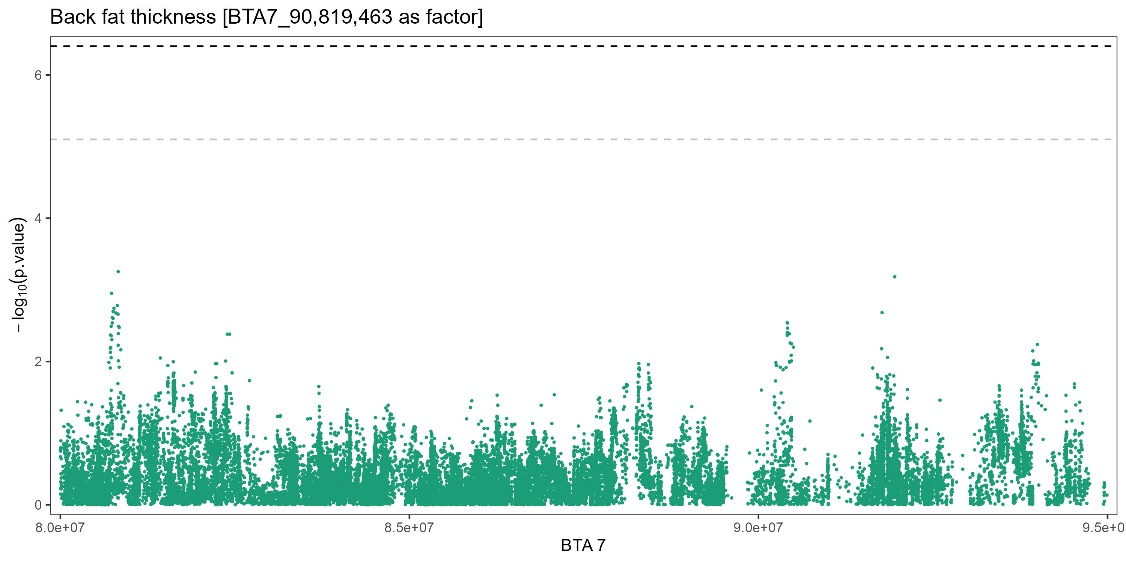


(d) Manhattan plot of GWAS for back fat thickness using IWGS variants (80–95 Mb on BTA7), without (left) and with (right) BTA7:90,819,463 as a fixed effect.

**Figure S8**: Manhattan plot of GWAS for carcass weight (a), rib-eye area (b), marbling score (c), and back fat thickness (d), using IWGS variants (80–95 Mb on BTA7), without (left) and with (right) BTA7:90,819,463 as a fixed effect.
